# Biomimetic PD-1-Functionalized Immunostimulatory Nanomedicine Enables STING Activation and Durable Antitumor Immunity in Hepatocellular Carcinoma

**DOI:** 10.1007/s40820-026-02320-7

**Published:** 2026-07-30

**Authors:** Yanbing Cao, Xinyi Lin, Bingchen Wu, Yang Li, Wenrui Zhu, Binxin Liu, Aixian Zheng, Lingjie Wu, Yanyang Wang, Xiaolong Liu, Ming Wu

**Affiliations:** 1 The United Innovation of Mengchao Hepatobiliary Technology Key Laboratory of Fujian Province, Mengchao Hepatobiliary Hospital of Fujian Medical University, Fuzhou, 350025 People’s Republic of China; 2 Department of Radiation Oncology, General Hospital of Ningxia Medical University, Yinchuan, 750004 People’s Republic of China; 3 The Liver Center of Fujian Province, Fujian Medical University, Fuzhou, 350025 People’s Republic of China; 4 Institute of Medical Sciences, General Hospital of Ningxia Medical University, Yinchuan, 750004 People’s Republic of China; 5 State Key Laboratory of Structural Chemistry & CAS Key Laboratory of Design and Assembly of Functional Nanostructures, Fujian Institute of Research on the Structure of Matter, Chinese Academy of Sciences, Fuzhou, 350002 People’s Republic of China

**Keywords:** Hepatocellular carcinoma, STING agonist, Photothermal therapy, Biomimetic nanomedicine, Immune checkpoint blockade

## Abstract

Hepatocellular carcinoma (HCC) responds poorly to immune checkpoint blockade, largely because of an immunologically cold tumor microenvironment (TME) characterized by deficient antigen presentation and impaired cytotoxic T cell responses. Analysis of numerous HCC clinical cohorts, including our institutional datasets, reveals that stimulator of interferon genes (STING) pathway activity is positively correlated with patient survival, enhanced antigen presentation capacity, and an immune-activated TME. However, achieving effective and controllable STING activation in immune-cold HCC tumors remains challenging. Here, we develop a biomimetic nanomedicine that integrates photothermal therapy (PTT), STING pathway activation, and immune checkpoint blockade to treat refractory HCC. A coordination nanomedicine MCI-NP is engineered by co-encapsulating the STING agonist MSA-2 and indocyanine green (ICG) via Cu^2+^-mediated chelation, enabling stabilized PTT-induced immunogenic cell death together with robust STING-driven innate immune activation. Further cloaked with PD-1-overexpressing cell membranes, MCI-NP@mPD-1 achieves an approximately fivefold increase in tumor accumulation compared with uncoated MCI-NP and enables localized PD-1/PD-L1 axis blockade. Following a single treatment, MCI-NP@mPD-1-based photothermal immunotherapy effectively suppresses primary tumor growth and significantly prolongs survival by remodeling the TME toward an immune-active state, while inducing durable systemic immune memory that effectively limited postoperative lung metastasis. Without introducing additional nanocarriers or excipients, MCI-NP@mPD-1 offers a promising therapeutic paradigm for photothermal immunotherapy of immune-cold HCC.

**Supplementary Information:**

The online version contains supplementary material available at 10.1007/s40820-026-02320-7.

## Highlights

- Clinical cohort analyses identify stimulator of interferon genes (STING) signaling as a key determinant of immune responsiveness in hepatocellular carcinoma (HCC), providing a data-driven rationale for nanomedicine design.
- A carrier-free coordination nanomedicine co-assembles MSA-2 and indocyanine green to couple photothermal-induced immunogenic cell death with STING activation, while PD-1 membrane camouflage enables ~fivefold tumor accumulation and localized checkpoint blockade.
- This integrated strategy converts immune-cold HCC into an immune-active state, achieving durable tumor control and systemic immune memory against metastasis.

**Supplementary Information:**

The online version contains supplementary material available at 10.1007/s40820-026-02320-7.

## Graphical abstract

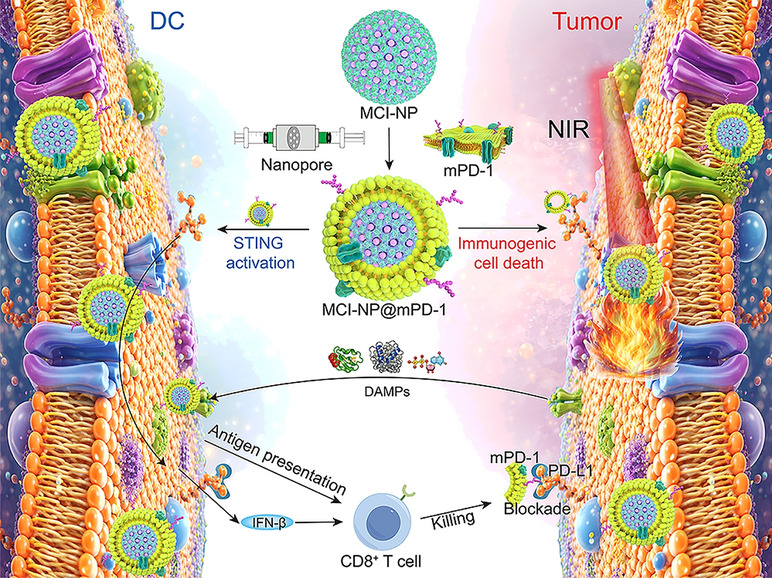

## Introduction

**Fig. 1:**
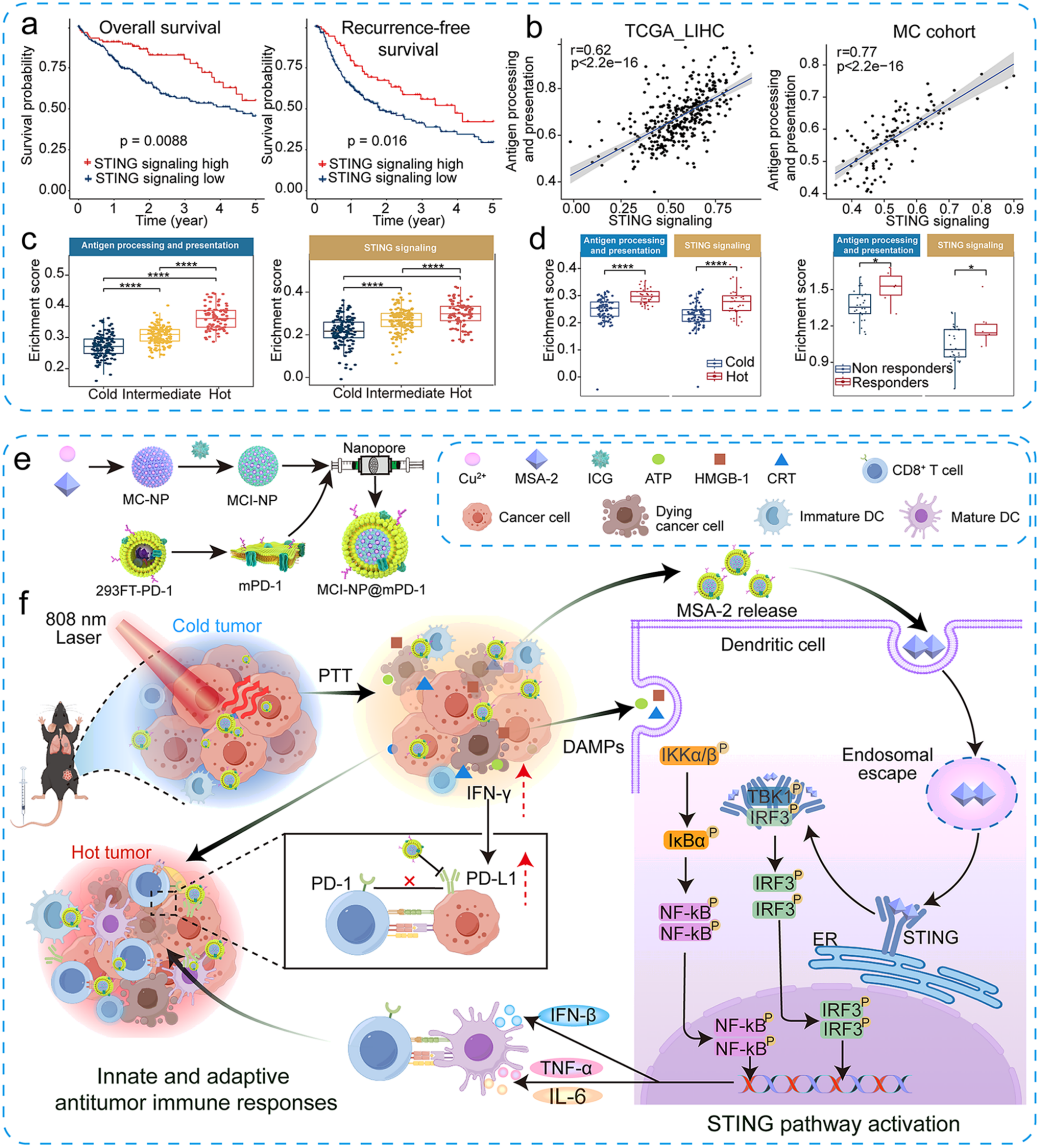
Clinical relevance of STING signaling in HCC and schematic illustration of the MCI-NP@mPD-1. **a** Kaplan–Meier analysis of 5-year overall survival and recurrence-free survival in HCC patients from the TCGA-LIHC cohort stratified by STING pathway activity. **b** Correlation analysis between STING signaling activity and “antigen processing and presentation-related gene signatures” in the TCGA-LIHC cohort and our institutional HCC (MC) cohort. **c** Enrichment scores of “antigen processing and presentation programs” and STING signaling across immune-cold, intermediate and hot HCC tumors in the TCGA cohort. **d** Enrichment scores of antigen processing and presentation programs and STING signaling in the MC cohort, stratified by immune phenotype (immune-hot vs immune-cold, left) and by clinical response to PD-1 blockade in PRJEB34724 cohort (responders vs non-responders, right). **e** Schematic illustration of the preparation of MCI-NP via Cu^2+^-bridged coordination assembly of ICG and MSA-2, followed by cell membrane camouflage to generate MCI-NP@mPD-1 using PD-1-overexpressing 293FT cell membranes. **f** Proposed mechanism of MCI-NP@mPD-1 in vivo: ICG-mediated photothermal conversion induces localized ICD with tumor antigen and DAMP release, while MSA-2 activates STING signaling, and mPD-1 enhances tumor accumulation and locally blocks the PD-1/PD-L1 axis, collectively reprogramming immune-cold HCC toward an immune-active state

Hepatocellular carcinoma (HCC) responds poorly to immune checkpoint blockade, with objective response rates generally below 20% [1, 2]. The limited efficacy is closely associated with the immunologically cold tumor microenvironment (TME), which is characterized by low tumor antigenicity, inefficient antigen presentation, and insufficient or dysfunctional tumor-specific cytotoxic T lymphocyte [3–5]. Consequently, effective immunotherapy for HCC requires strategies that restore antigen presentation capacity and reinstate productive antitumor T cell immunity. Antigen presentation and T cell priming are initiated by innate immune sensing, among which the stimulator of interferon genes (STING) pathway plays a central role. STING activation induces type I interferon signaling, promotes dendritic cell (DC) maturation, and facilitates antigen cross-presentation, thereby coupling innate danger sensing to downstream adaptive immune responses [6–8]. As expected, analyses of the Cancer Genome Atlas (TCGA) dataset revealed that elevated STING pathway activity in HCC was associated with better overall and recurrence-free survival (Fig. 1a). Across both the TCGA cohort and our institutional MC cohort, STING signaling showed a strong positive correlation with gene signatures related to antigen processing and presentation (Fig. 1b). Moreover, immune-hot HCC tumors were characterized by higher enrichment of both STING signaling and antigen presentation programs compared with immune-cold tumors, a trend consistently observed across both datasets (Figs. 1c, d, and S1). Notably, in a clinical HCC immunotherapy cohort (PRJEB34724), responders to PD-1 blockade also exhibited higher enrichment of antigen presentation programs and STING signaling (Fig. 1d). Together, these observations underscore the relevance of STING activity to effective antigen presentation and immune-inflamed tumor states in HCC, supporting its potential as a rational and actionable immunotherapeutic target.

Despite the strong immunological and clinical associations of STING signaling in HCC, achieving effective and controllable STING activation within tumors remains challenging [9]. Currently available STING agonists include cyclic dinucleotides (CDNs) and next-generation small-molecules. Among them, MSA-2 is particularly attractive due to its pharmacological tractability, oral/systemic bioavailability, and its ability to activate STING without intratumoral injection [9–11]. However, in immune-cold solid tumors such as HCC, STING agonist monotherapy frequently fails to induce robust or durable immune activation, largely owing to insufficient endogenous danger signals and poor antigen availability within the TME [6, 12]. Additionally, nonspecific STING activation may provoke systemic inflammation, raising safety concerns that hinder clinical translation [13]. Therefore, effective STING-based immunotherapy requires strategies that are not only capable of achieving tumor-localized and efficient STING activation but also integrating complementary modalities, particularly immunogenic cell death (ICD) inducers that enhance tumor immunogenicity and antigen availability to improve overall therapeutic efficacy.

Photothermal therapy (PTT) can act as a potent ICD inducer, promoting the release of tumor-associated antigens and damage-associated molecular patterns (DAMPs) that trigger innate immune activation and facilitate subsequent adaptive immune responses [14]. PTT-induced cellular stress and DNA damage may increase the availability of cytosolic DNA, facilitating the cGAS-STING axis engagement and type I interferon-dominated inflammatory signaling [15, 16]. However, STING activation elicited by PTT alone is typically limited in magnitude and duration and is frequently inadequate to fully reprogram the immunosuppressive landscape of immune-cold tumors such as HCC or sustain durable antitumor immunity [17]. Consequently, combining PTT with STING agonists represents a mechanistically rational strategy, whereby PTT-induced ICD supplies an in situ source of tumor antigens and danger signals, while exogenous STING agonists amplify and sustain pathway activation [18], thereby cooperatively enhancing downstream adaptive immune responses in immune-cold HCC. Indocyanine green (ICG), an FDA-approved near-infrared (NIR) fluorophore, is widely used as a photothermal agent owing to its established clinical safety and photothermal performance [19–21]. However, the broader application of ICG in PTT is limited by several intrinsic drawbacks, including poor aqueous stability with a strong tendency to aggregation, rapid systemic clearance resulting in a short circulation half-life, insufficient tumor accumulation and retention, and susceptibility to photobleaching or photodegradation under repeated irradiation. Collectively, these limitations compromise heating efficiency and therapeutic reproducibility, underscoring the need for rational delivery strategies to improve the therapeutic reliability of ICG-based photothermal systems.

Nanomedicine-based delivery systems provide an effective strategy for tumor-targeted delivery of STING agonists [22, 23]. In addition, accumulating evidence [21, 24, 25], including our previous work [19], indicates that nanodelivery systems can protect ICG by physically confining and dispersing the dye homogeneously, thereby limiting its exposure to aqueous and oxidative environments, reducing photobleaching and photodegradation under irradiation, and ultimately enhancing photothermal stability. Herein, we designed a coordination nanomedicine (denoted as MCI-NP) by co-incorporating MSA-2 and ICG through Cu^2+^-mediated chelation (Fig. 1e). MCI-NP can induce tumor ICD via PTT while concomitantly activating the STING pathway to suppress tumor growth (Fig. 1f), an effect that was markedly attenuated in STING-knockout (STING-KO) mice. To further improve systemic performance and tumor-selective accumulation following intravenous administration, we cloaked MCI-NP with our previously developed genetically engineered cell membrane overexpressing PD-1 (mPD-1) [26–29], generating a biomimetic nanomedicine termed MCI-NP@mPD-1. In addition to enhancing tumor targeting through PD-1/PD-L1 interactions, membrane-displayed PD-1 can function as a decoy receptor that competitively sequesters PD-L1, thereby conferring localized immune checkpoint blockade activity. Collectively, MCI-NP@mPD-1 effectively activates the host immune system, resulting in complete tumor regression in Hepa1-6 tumor-bearing mice and markedly suppressing tumor recurrence and metastasis. Thus, the designed MCI-NP@mPD-1 integrates tumor-localized photothermal ablation, STING-mediated innate immune amplification, and local checkpoint modulation, providing a rational and synergistic photothermal-immunotherapeutic framework for overcoming immune resistance in immune-cold HCC.

## Experimental Section

All animal experiments were conducted in strict accordance with institutional guidelines and protocols approved by the Ethics Committee of Mengchao Hepatobiliary Hospital of Fujian Medical University (MCHH-AEC-2023-070). The raw RNA-sequencing data generated from the mouse model in this study have been deposited in the Genome Sequence Archive (GSA; https://ngdc.cncb.ac.cn/gsa) under accession number CRA042678. Detailed experimental procedures are provided in the Supporting Information.

## Results and Discussion

### Fabrication and Characterization of MCI-NP

**Fig. 2:**
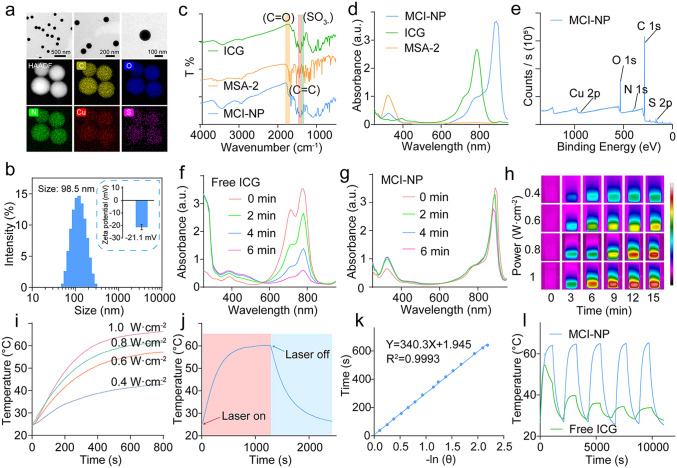
Fabrication and characterization of MCI-NP.** a** TEM image of MCI-NP and corresponding HAADF-STEM elemental mapping showing the spatial distribution of C, O, N, Cu and S. **b** DLS analysis of MCI-NP, showing hydrodynamic size distribution and zeta potential. **c** FT-IR spectra and **d** UV–vis absorption spectra of free ICG, free MSA-2, and MCI-NP. **e** XPS survey spectrum of MCI-NP. Time-dependent UV–Vis absorbance changes of** f** free ICG (100 μg mL^−1^) and **g** MCI-NP (200 μg mL^−1^) under continuous 808 nm laser irradiation (0.8 W cm^−2^). **h** Infrared thermal images and **i** the corresponding temperature–time profiles of MCI-NP aqueous solutions (100 μg mL^−1^) under 808 nm laser irradiation at different power densities (0.4–1.0 W cm^−2^). **j** Heating and cooling curves of MCI-NP solutions (100 μg mL^−1^) recorded under 808 nm laser irradiation (0.8 W cm^−2^) for photothermal conversion efficiency calculation. **k** Linear fitting of time versus − ln(θ) derived from the cooling curve used to determine photothermal conversion efficiency. **l** Photothermal stability of free ICG and MCI-NP (100 μg mL^−1^) over five consecutive laser on/off cycles under 808 nm irradiation (0.8 W cm^−2^)

The designed MCI-NP was fabricated through a Cu^2+^-bridged coordination strategy to co-assemble ICG and the small-molecule STING agonist MSA-2 into a unified nanostructure. Transmission electron microscopy (TEM) images showed that MCI-NP exhibited a uniform and well-dispersed nanosphere morphology (Fig. 2a). High-angle annular dark-field scanning transmission electron microscopy (HAADF-STEM) elemental mapping further revealed a homogeneous spatial distribution of Cu together with the organic elements (C, O, N, and S) within individual nanoparticles (Fig. 2a). Dynamic light scattering (DLS) analysis indicated that MCI-NP possessed a hydrodynamic diameter of 98.5 nm with a narrow size distribution, suggesting good colloidal uniformity (Fig. 2b). In addition, the zeta potential of MCI-NP was measured to be −21.1 mV, reflecting a moderately negative surface charge (Fig. 2b). Following characterization of morphology and colloidal properties, Fourier transform infrared (FT-IR) spectroscopy was employed to analyze the chemical composition of MCI-NP (Fig. 2c). A characteristic band at 1045 cm^−1^ corresponding to the symmetric stretching of sulfonate groups (–SO_3_^−^) from ICG was observed, together with MSA-2-associated bands at 1650 cm^−1^ (C = O stretching) and 1605 cm^−1^ (aromatic C = C vibration) [30]. Ultraviolet–visible (UV–Vis) absorption spectroscopy further verified the co-assembly of ICG and MSA-2 within MCI-NP (Fig. 2d). Characteristic absorption peaks at approximately 780 and 320 nm, corresponding to ICG and MSA-2, respectively, were simultaneously observed [30, 31]. Compared with free ICG, the ICG absorption peak in MCI-NP showed a slight redshift, attributable to intermolecular π–π stacking and hydrophobic interactions induced by nanostructure formation [30]. These spectral features support the effective co-loading of ICG and MSA-2 within a composite nanostructure rather than a simple physical mixture. X-ray photoelectron spectroscopy (XPS) analysis of MCI-NP confirmed the presence of Cu, O, N, C, and S elements (Fig. 2e), consistent with the HAADF-STEM elemental mapping results (Fig. 2a). To elucidate the interactions underlying the self-assembly of MCI-NP, molecular docking, chemical disruption experiments, and high-resolution XPS analyses were performed (Fig. S2). The simulation suggested multiple intermolecular interactions among the components, including metal coordination, hydrophobic interactions, electrostatic interactions, and π-related non-covalent interactions (Fig. S2a). To further validate these interactions, various chemical agents, including SDS, EDTA, NaCl, and urea, were introduced into the MCI-NP to selectively disrupt these forces. Notably, among all tested agents, only EDTA induced a pronounced shift in the absorption spectrum of MCI-NP, suggesting disruption of the nanoparticle structure through chelation of Cu^2+^, whereas SDS, NaCl, and urea produced negligible effects (Fig. S2b). Moreover, high-resolution O 1*s* XPS spectra revealed evident changes in the Cu–O-related chemical environments after nanoparticle formation, providing additional evidence for the existence of Cu–O coordination interactions (Fig. S2c). Collectively, these results support that Cu^2+^-mediated coordination serves as the primary driving force for nanoparticle assembly, while hydrophobic, electrostatic, and π-related interactions further contribute to structural stabilization. To further evaluate the composition of MCI-NP, the loading contents of ICG and MSA-2 were quantified by HPLC analysis using standard calibration curves (Fig. S3a). The resulting MCI-NP exhibited loading contents of 32 wt% for ICG and 30 wt% for MSA-2. As MCI-NP is a carrier-free nanostructure composed solely of ICG, MSA-2, and Cu^2+^, the Cu content was estimated to be approximately 38 wt%. In addition, in vitro release studies revealed a pronounced pH-dependent release behavior, with both ICG and MSA-2 being preferentially released under mildly acidic conditions (pH 5.5) compared with physiological pH 7.4 (Fig. S3b), supporting TME-responsive drug release. Taken together, all these results demonstrate the successful assembly of a structurally stable MCI-NP nanomedicine.

The photothermal performance of MCI-NP was next evaluated under 808 nm laser irradiation (0.8 W cm^−2^). Upon continuous irradiation, free ICG exhibited a pronounced decrease in absorbance over time, indicating limited photostability (Fig. 2f), whereas MCI-NP maintained stable absorption profiles under identical conditions (Fig. 2g). To investigate the origin of this enhanced photostability, a physical mixture of Cu^2+^, MSA-2, and ICG was prepared and evaluated under the same conditions (Fig. S4a). Notably, the mixture displayed rapid photobleaching behavior comparable to that of free ICG, whereas MCI-NP exhibited minimal photobleaching, suggesting that the improved photostability primarily arises from nanoparticle assembly rather than protection by any individual component. Infrared thermal imaging and temperature–time profiles revealed that MCI-NP solutions exhibited a robust, power density-dependent photothermal response upon laser irradiation (Fig. 2h, i). MCI-NP induced temperature elevations of 24.6 °C (100 μg mL^−1^, 0.8 W cm^−2^) and 20.2 °C (400 μg mL^−1^, 0.33 W cm^−2^), markedly exceeding the temperature increase observed in the PBS control (Fig. S2c, d). The photothermal conversion efficiency of MCI-NP was further quantified based on the Roper method [32] by analyzing the heating and cooling curves (Fig. 2j, k), yielding a photothermal conversion efficiency of 52.3%, substantially higher than that reported for free ICG (20%) [33]. In addition, MCI-NP exhibited stable photothermal performance over five consecutive heating–cooling cycles without obvious attenuation in temperature elevation, whereas free ICG showed a pronounced decline in photothermal efficiency (Fig. 2l). Similarly, the Cu^2+^/MSA-2/ICG mixture showed inferior photothermal stability compared with MCI-NP during repeated heating–cooling cycles (Fig. S4b), highlighting the importance of nanoparticle assembly in maintaining photothermal performance. Overall, these results demonstrate that MCI-NP enables efficient and stable photothermal heat generation under physiologically relevant irradiation conditions, supporting its suitability as a photothermal agent for tumor ablation applications.

### In Vitro Photothermal Effects and Immunogenic Activation Induced by MCI-NP

**Fig. 3:**
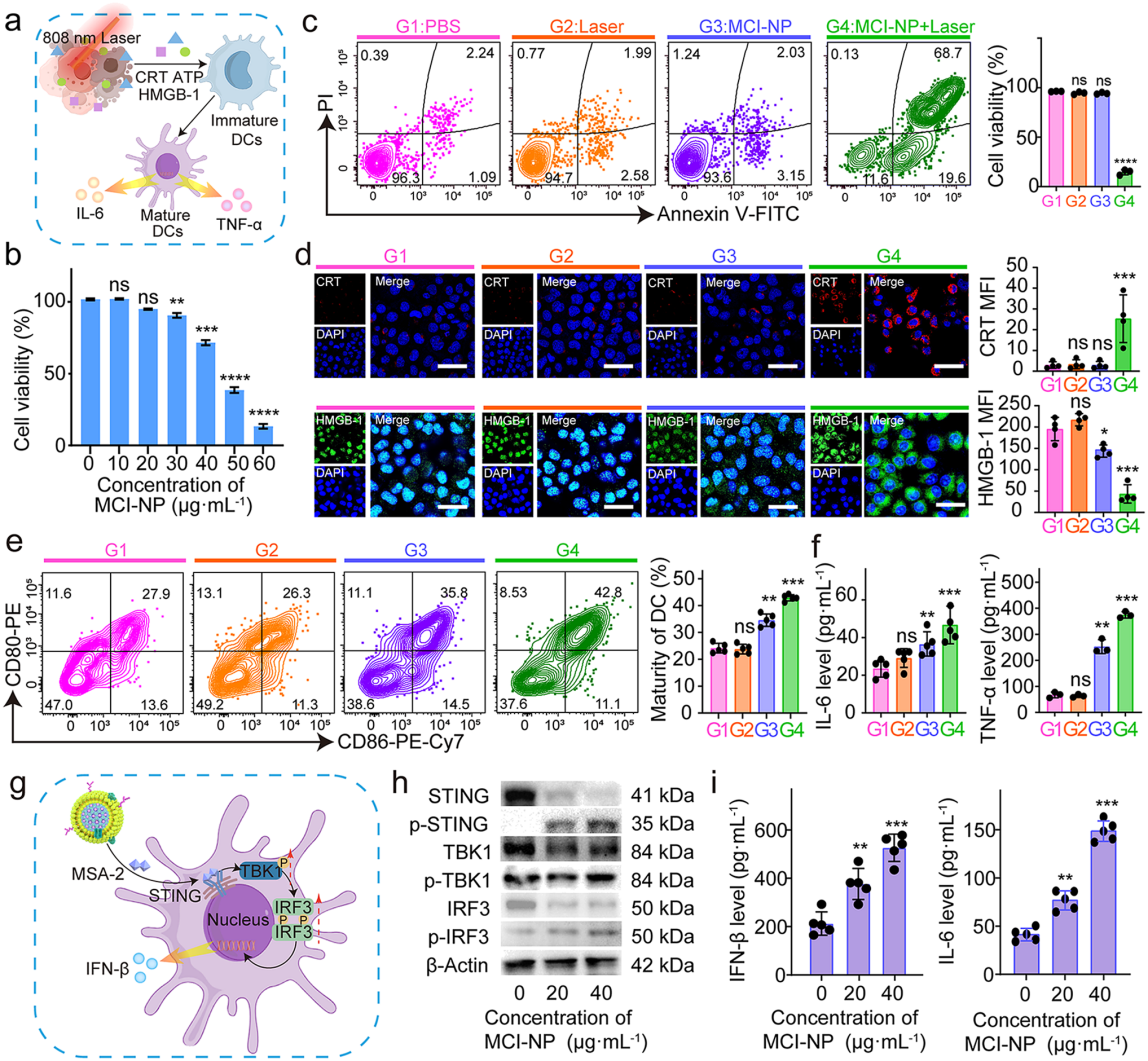
MCI-NP-mediated photothermal tumor cell killing induces immunogenic cell death and activates innate immune responses in vitro. Unless otherwise specified, treatment groups were defined as follows: G1, PBS; G2, laser irradiation alone; G3, MCI-NP alone; G4, MCI-NP combined with laser irradiation. **a** Schematic illustration of MCI-NP-mediated photothermal tumor cell death and associated immunogenic activation.** b** Cell viability of Hepa1-6 cells treated with increasing concentrations of MCI-NP under 808 nm laser irradiation (0.8 W cm^−2^, 10 min), assessed by CCK-8 assay (n = 4). **c** Flow cytometric analysis of apoptosis in Hepa1-6 cells after different treatments. Representative Annexin V/PI dot plots (left) and corresponding quantification of alive cell populations (right) are shown (n = 3). **d** Immunofluorescence staining of CRT and HMGB-1 in Hepa1-6 cells after different treatments. Cells were stained for CRT (red) and HMGB-1 (green), with nuclei counterstained with DAPI (blue). Scale bars, 50 μm. **e** Flow cytometric analysis of DC maturation after incubation with conditioned media from Hepa1-6 cells subjected to different treatments. Representative density plots showing CD80 and CD86 expression on BMDCs (left) and corresponding quantification of CD80^+^ CD86^+^ DC populations (right) are shown (CD11c^+^ gate) (n = 5).** f** ELISA quantification of IL-6 and TNF-*α* levels in culture supernatants of BMDCs incubated with conditioned media from differently treated Hepa1-6 cells (n = 5). **g** Schematic overview of STING pathway activation in BMDCs following MCI-NP treatment, highlighting downstream TBK1 and IRF3 signaling.** h** Western blot analysis of STING pathway activation in BMDCs following incubation with different concentrations of MCI-NP, showing total and phosphorylated STING (STING/p-STING), TBK1 (TBK1/p-TBK1), and IRF3 (IRF3/p-IRF3). **i** ELISA quantification of IFN-β and IL-6 levels in culture supernatants of BMDCs following incubation with different concentrations of MCI-NP (n = 5). Statistics: One-way ANOVA for **b, c, d, e, f** and **i**, compared with the PBS control group. Data are presented as mean ± SD. Significance levels: *p < 0.05, **p < 0.01, ***p < 0.001, ****p < 0.0001

To ensure the feasibility of subsequent photothermal evaluation at the cellular level, the cellular uptake behavior and cytocompatibility of MCI-NP were first examined. Confocal laser scanning microscopy (CLSM) confirmed efficient and time-dependent internalization of MCI-NP in Hepa1-6 cells, reaching uptake saturation within 4h (Fig. S5a). Similar efficient internalization was observed in DC2.4 cells, indicating that MCI-NP can be readily taken up by both tumor cells and antigen-presenting cells (Fig. S5b). CCK-8 assays showed negligible cytotoxicity toward both Hepa1-6 tumor cells and normal hepatocyte CL2 cells, even at concentrations up to 400 μg mL^−1^ (Fig. S5c). Under equivalent Cu^2+^ concentrations, free Cu^2+^ caused significantly greater cytotoxicity than MCI-NP, indicating that nanoparticle assembly effectively mitigated the intrinsic cytotoxicity associated with Cu^2+^ (Fig. S5d). These results collectively demonstrate the good cytocompatibility of MCI-NP. Based on these observations, we next evaluated the cellular biological effects of MCI-NP-mediated PTT, including tumor cell ablation and the induction of immunogenic responses, as schematically illustrated in Fig. 3a. Upon exposure to 808 nm laser irradiation (0.8 W cm^−2^, 10 min), Hepa1-6 cells treated with MCI-NP exhibited a pronounced and concentration-dependent reduction in cell viability (Fig. 3b). At a concentration of 60 μg mL^−1^, MCI-NP combined with laser irradiation reduced cell viability below 20%, indicating effective photothermal ablation of tumor cells. Consistently, live/dead staining revealed extensive cell death exclusively in the MCI-NP + Laser group, while minimal cell death was detected in the PBS, MCI-NP alone, or laser-only control groups (Fig. S5e). Flow cytometric analysis further showed a marked increase in apoptotic cell populations following MCI-NP-mediated PTT (85.4%), confirming that laser-activated MCI-NP effectively induces tumor cell death (Fig. 3c).

Having established the photothermal cytotoxicity of MCI-NP, we next investigated whether MCI-NP-mediated PTT induces ICD, a critical prerequisite for antitumor immune activation. Hallmark damage-associated molecular patterns (DAMPs), including calreticulin (CRT) exposure and high-mobility group box 1 (HMGB-1) release [34], were examined following photothermal treatment. Immunofluorescence analysis showed that MCI-NP combined with laser irradiation induced robust CRT exposure on the tumor cell surface, whereas CRT signals were weak or scarcely detectable in all control groups (Fig. 3d). Consistently, HMGB-1 exhibited a clear redistribution from the nucleus to the cytoplasmic and extracellular compartments after MCI-NP-mediated PTT, which is indicative of HMGB-1 release (Fig. 3d). In addition, extracellular ATP release was significantly increased after PTT, further supporting the induction of ICD (Fig. S5f). Together, these findings demonstrate that MCI-NP-mediated PTT triggers characteristic ICD-associated DAMP exposure and release.

Having demonstrated that MCI-NP-mediated PTT induces ICD, we next examined whether tumor cell-derived immunogenic signals released upon PTT could promote DC maturation. Bone marrow-derived DCs (BMDCs) were incubated with conditioned media from Hepa1-6 cells subjected to different treatments, and DC maturation was assessed by flow cytometric analysis of CD80 and CD86 expression. Compared with control groups, supernatants from the MCI-NP + Laser group markedly increased the proportion of CD80^+^ CD86^+^ DCs to 42.9%, substantially higher than that observed for PBS (23.8%), Laser (24.3%), or MCI-NP alone (34.6%) groups (Figs. 3e and S6). Notably, conditioned media from MCI-NP-treated cells without laser irradiation also induced a modest increase in DC maturation, consistent with the intrinsic immunostimulatory activity of the STING agonist MSA-2. To further investigate the contribution of individual components to DC maturation, BMDCs were directly incubated with Cu^2+^, MSA-2/Cu^2+^, ICG/Cu^2+^, or MCI-NP (Fig. S7). The results showed that Cu^2+^ (12.9%) and ICG/Cu^2+^ (14.4%) induced only minimal increases in CD80^+^ CD86^+^ DCs compared with the control group (10.1%), while both MSA-2/Cu^2+^ (24.0%) and MCI-NP (25.5%) substantially increased the proportion of CD80^+^ CD86^+^ DCs. These results suggest that the DC maturation activity of MCI-NP is largely attributable to the STING agonist MSA-2, whereas Cu^2+^ and ICG contribute minimally to this process. In addition to phenotypic maturation, DC functional activation was further evaluated by measuring inflammatory cytokine secretion. ELISA analysis of DC culture supernatants revealed that conditioned media from both MCI-NP and MCI-NP + Laser-treated tumor cells significantly increased the production of IL-6 and TNF-α compared with control groups (Fig. 3f). These results indicate that MCI-NP-mediated PTT generates an immunogenic tumor cell milieu capable of effectively promoting DC maturation and pro-inflammatory cytokine secretion, thereby functionally linking ICD to subsequent immune activation.

Activation of the STING pathway represents another key immunological function of the MCI-NP (Fig. 3g). To assess STING signaling activation, we first examined key downstream proteins in DCs following direct incubation with MCI-NP at different concentrations by Western blot analysis. Compared with control groups, MCI-NP treatment markedly increased the phosphorylation levels of STING (Fig. 3h), TBK1, and IRF3, indicating effective activation of the STING signaling cascade [35, 36]. Consistent with these results, ELISA assays revealed significantly elevated secretion of type I interferon (IFN-β) and the pro-inflammatory cytokine IL-6 in the culture supernatant after MCI-NP treatment (Fig. 3i). Notably, IFN-β secretion increased by approximately 1.3-fold as the MCI-NP concentration increased from 20 to 40 μg mL^−1^, while IL-6 levels exhibited an approximately 1.96-fold increase over the same concentration range. Similarly, MCI-NP also activated the STING pathway in Hepa1-6 tumor cells, as evidenced by increased IFN-β and IL-6 secretion measured by ELISA (Fig. S8). Collectively, these results demonstrate that MCI-NP effectively activates the cGAS-STING pathway in DCs and tumor cells, thereby providing a molecular basis for enhanced DC maturation and subsequent antitumor immune responses.

### In Vivo Photothermal-Immunotherapeutic Efficacy of MCI-NP

**Fig. 4:**
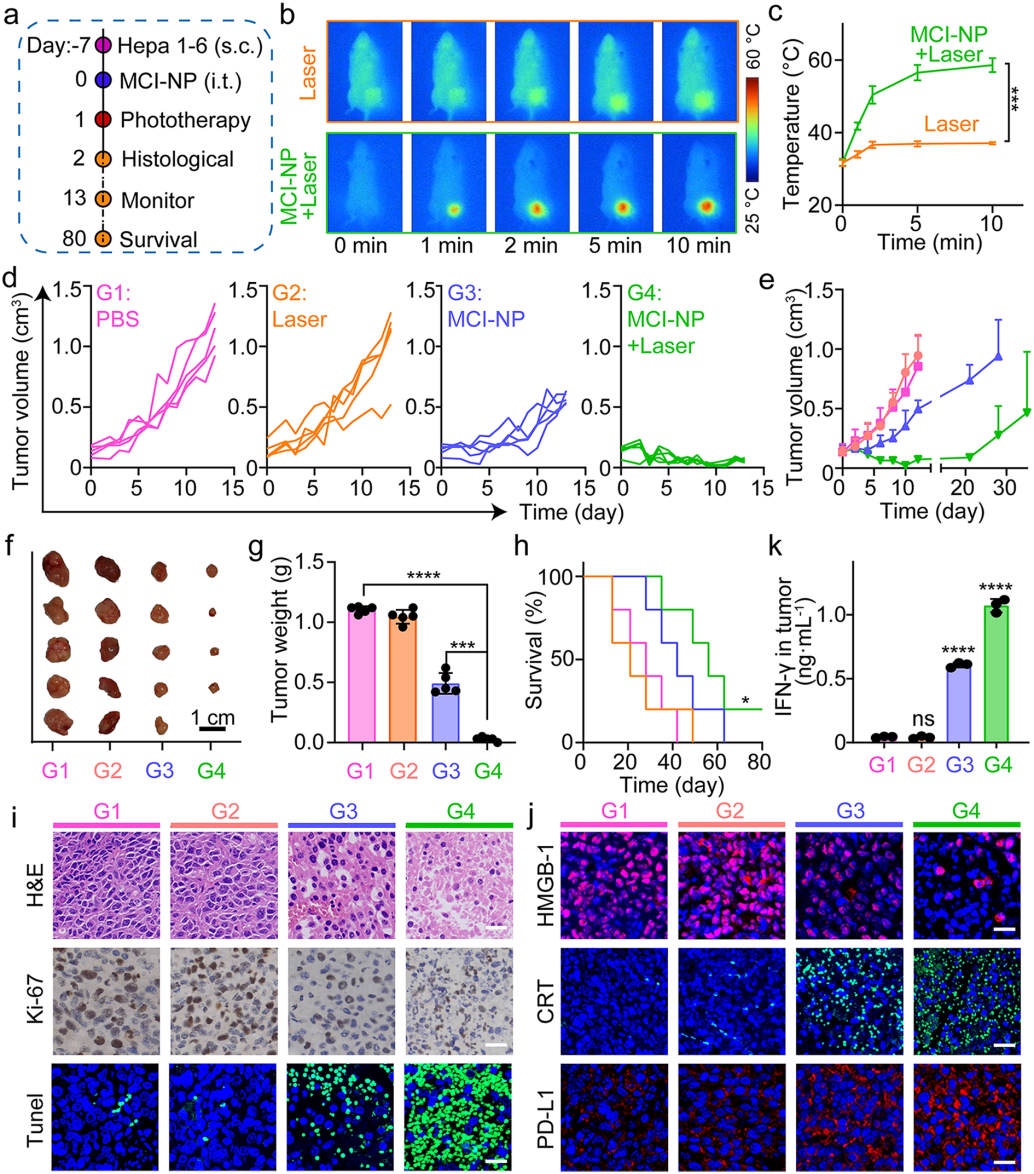
In vivo photothermal-immunotherapeutic efficacy of MCI-NP in a Hepa1-6 subcutaneous tumor model. Treatment groups: G1, PBS; G2, laser irradiation alone; G3, MCI-NP alone; G4, MCI-NP combined with laser irradiation. **a** Schematic illustration of the in vivo treatment schedule for MCI-NP-mediated PTT in Hepa1-6 subcutaneous tumor-bearing mice. Tumors received intratumoral injection of MCI-NP or PBS, followed by 808 nm laser irradiation 24 h later (0.8 W cm^−2^, 10 min). **b** Representative infrared thermal images of tumor-bearing mice after intratumoral injection and subsequent laser irradiation (0.8 W cm.^−2^, 10 min). **c** Corresponding temperature elevation curves at the tumor site during laser irradiation. **d** Tumor growth curves of individual mice receiving different treatments. **e** Average tumor volume growth curves of mice in each treatment group. **f** Representative photographs of excised tumors at the treatment endpoint (Day 13). **g** Tumor weights measured at the treatment endpoint. **h** Kaplan–Meier survival curves of mice following different treatments. **i** Histopathological analysis of tumor tissues collected on day 2, including H&E staining, Ki67 immunohistochemistry, and TUNEL staining. Scale bars, 50 µm. **j** Immunofluorescence staining of HMGB-1, CRT, and PD-L1 in tumor sections after different treatments. Scale bars, 50 µm. **k** ELISA quantification of intratumoral IFN-γ levels after treatment. Statistics: Two-tailed unpaired *t*-test for **c**; log-rank (Mantel-Cox) test for **h**; One-way ANOVA for **k**. Data are presented as mean ± SD. Significance levels: *p < 0.05, ***p < 0.001, ****p < 0.0001

The antitumor efficacy of MCI-NP-mediated PTT was next evaluated in a Hepa1-6 subcutaneous tumor model following the treatment scheme shown in Fig. 4a. Prior to therapeutic assessment, the in vivo photothermal performance of MCI-NP was examined by infrared thermal imaging after intratumoral administration and subsequent 808 nm laser irradiation (0.8 W cm^−2^, 10 min). Whereas laser irradiation alone resulted in only a minimal temperature increase at the tumor site (up to 37.1 °C), tumors treated with MCI-NP exhibited a rapid and pronounced temperature elevation, reaching approximately 58.6 °C, sufficient for effective thermal ablation, thereby confirming the efficient photothermal conversion capability of MCI-NP (Fig. 4b, c).

Mice were then randomly assigned to four treatment groups: PBS (G1), Laser (G2), MCI-NP (G3), and MCI-NP + Laser (G4). Tumor growth was not significantly affected by laser irradiation alone, while MCI-NP treatment produced a moderate delay in tumor progression compared with PBS or Laser controls (Fig. 4d, e). In contrast, MCI-NP combined with laser irradiation (MCI-NP + Laser) resulted in marked tumor growth suppression, demonstrating a pronounced therapeutic benefit of photothermal ablation. Consistent with these findings, excised tumors from the MCI-NP + Laser group were substantially smaller and lighter than those from control groups at the treatment endpoint (Day 13) (Fig. 4f, g). Survival analysis further revealed that MCI-NP-mediated PTT significantly prolonged overall survival relative to control treatments (Fig. 4h). No apparent body weight loss was observed during the early observation period (Fig. S9a), indicating acceptable treatment tolerability.

To further evaluate the in vivo effects of MCI-NP-mediated PTT, tumor tissues were collected on day 2 for histopathological analysis. H&E staining revealed extensive tumor tissue damage in the MCI-NP + Laser group, accompanied by markedly reduced Ki67-positive proliferating cells and increased TUNEL-positive apoptotic cells (Figs. 4i and S10). In contrast, tumors from the PBS and Laser groups retained intact morphology and high proliferative activity, while the MCI-NP group showed only moderate tissue damage. Immunofluorescence analysis further demonstrated decreased intracellular HMGB-1 signals and enhanced CRT exposure in tumors treated with MCI-NP or MCI-NP + Laser (Figs. 4j and S10), indicating the induction of ICD in vivo. Consistent with STING pathway activation, intratumoral IFN-*β* levels were significantly elevated following MCI-NP-based treatments (Fig. S9b). Notably, the superior therapeutic efficacy observed in the MCI-NP + Laser group was associated with simultaneous induction of ICD and STING signaling in vivo. Given that ICD promotes the release of tumor-associated antigens and danger signals, whereas STING activation drives type I interferon production and innate immune activation [10, 12], these findings suggest that photothermal ablation and STING agonism may cooperatively enhance antitumor immune responses within the tumor microenvironment. Despite the pronounced early antitumor effects, tumors in the MCI-NP + Laser group eventually recurred. To investigate the underlying mechanism of recurrence, we analyzed the tumor microenvironment and observed markedly elevated IFN-*γ* levels (Fig. 4k). Consistent with the known role of IFN-γ in regulating PD-L1, we also detected concomitant upregulation of PD-L1 expression in these recurrent tumors (Fig. 4j). Notably, IFN-γ treatment directly induced PD-L1 upregulation in Hepa1-6 cells in vitro (Fig. S11), supporting the interpretation that the increased PD-L1 reflects a compensatory adaptive immune resistance mechanism triggered by enhanced antitumor immune activation [37]. These findings suggest that, while MCI-NP-mediated PTT effectively stimulates antitumor immunity, concomitant activation of the PD-1/PD-L1 axis may limit the durability of therapeutic responses. Therefore, combining STING-mediated immune activation with immune checkpoint blockade may counteract this adaptive resistance mechanism and sustain antitumor T cell activity.

Given the antitumor activity of MCI-NP observed even in the absence of laser irradiation, we next investigated whether this therapeutic effect is dependent on activation of the STING pathway by the encapsulated agonist MSA-2. The Hepa1-6 subcutaneous tumor model was therefore established in STING-knockout (STING-KO) mice. In contrast to wild-type mice, intratumoral administration of MCI-NP failed to suppress tumor growth in STING-KO mice, with no significant differences in tumor volume or tumor weight compared with PBS-treated group (Fig. S12). These findings indicate that the antitumor efficacy of MCI-NP is critically dependent on the intact STING signaling pathway, rather than direct cytotoxic or photothermal effects alone.

### Fabrication and Characterization of PD-1 Membrane-Camouflaged MCI-NP (MCI-NP@mPD-1)

Although intratumoral administration of MCI-NP elicited effective local photothermal ablation and STING-driven immune activation, tumor regrowth at later stages was accompanied by elevated PD-L1 expression, suggesting adaptive immune escape. To concurrently counteract PD-1/PD-L1-mediated immunosuppression and enable a transition toward systemic delivery with improved in vivo stability and tumor accumulation, we generated PD-1-overexpressing 293FT cells via genetic engineering and employed their plasma membranes to biomimetically cloak MCI-NP, yielding a PD-1 membrane-camouflaged nanoplatform (MCI-NP@mPD-1). This design integrates immune checkpoint blockade with enhanced circulation stability and tumor targeting, providing a rational strategy to improve the durability and translational potential of MCI-NP-based photothermal immunotherapy.

**Fig. 5:**
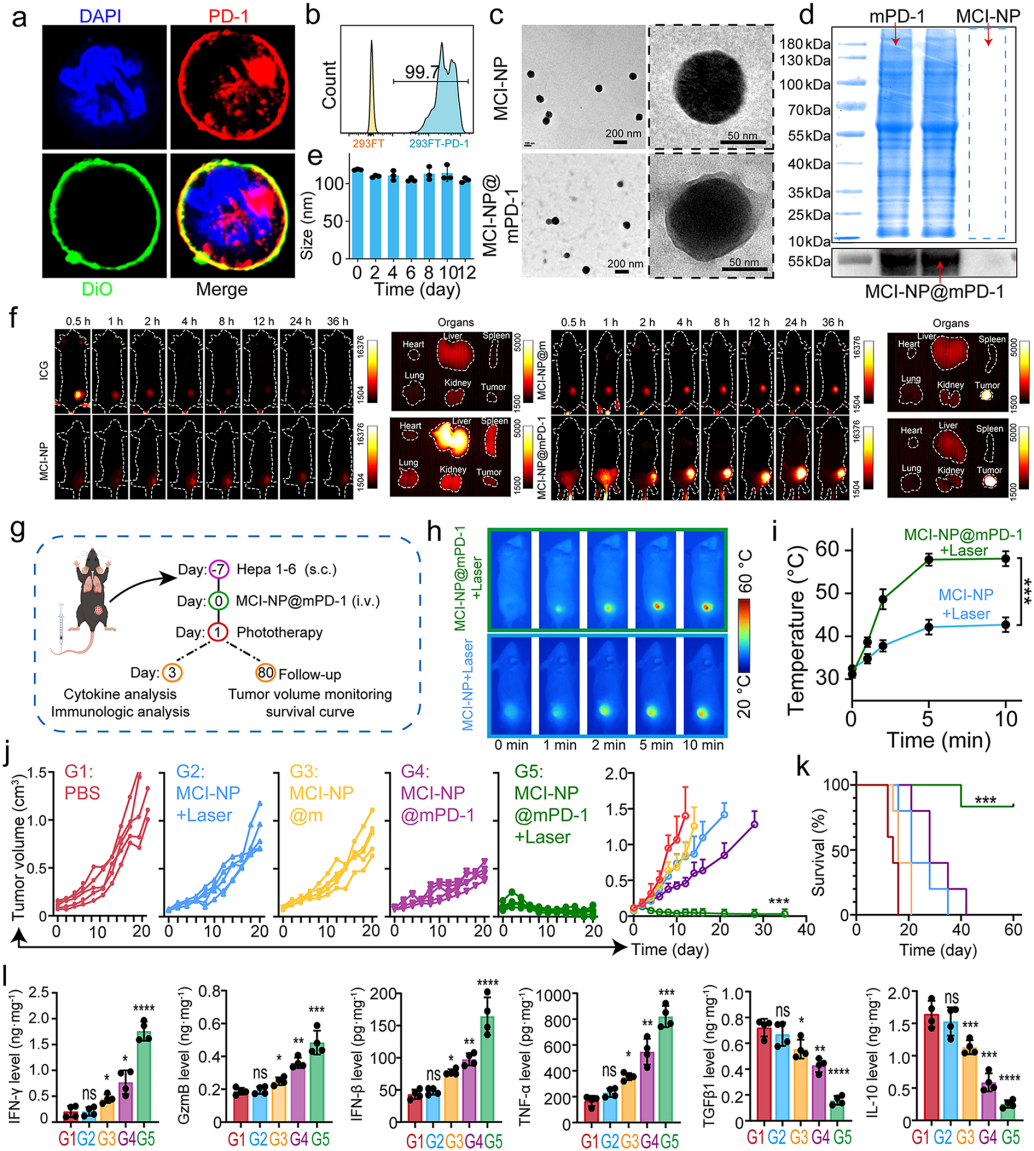
Engineering, characterization, in vivo performance, and immunomodulatory effects of MCI-NP@mPD-1. **a** CLSM images of genetically engineered PD-1-overexpressing 293FT cells (293FT-PD-1). **b** Flow cytometric analysis of PD-1 expression on 293FT-PD-1 cells. **c** TEM images of MCI-NP and MCI-NP@mPD-1. **d** Coomassie brilliant blue staining (top) and Western blot analysis (bottom) of isolated mPD-1 membranes, MCI-NP, and MCI-NP@mPD-1. **e** Colloidal stability of MCI-NP@mPD-1 monitored by DLS in medium containing 10% fetal bovine serum over 12 days. **f** In vivo fluorescence imaging of tumor-bearing mice following intravenous administration of free ICG, MCI-NP, MCI-NP@m, or MCI-NP@mPD-1, together with ex vivo fluorescence imaging of major organs (heart, liver, spleen, lung, and kidney) and tumors harvested at 24 h post-injection. **g** Schematic illustration of the in vivo treatment protocol for MCI-NP@mPD-1-mediated photothermal immunotherapy in the Hepa1-6 subcutaneous tumor model. Mice received intravenous administration of the indicated formulations, followed by 808 nm laser irradiation 24 h post-injection.** h** Infrared thermal images and **i** corresponding temperature profiles of tumors during 808 nm laser irradiation (0.8 W cm.^−2^, 10 min) 24 h after intravenous administration of the indicated formulations. **j** Tumor growth curves of mice under different treatment conditions, displaying tumor volume progression of individual animals as well as the corresponding mean tumor growth curves for each group. **k** Kaplan–Meier survival curves of treated mice. **l** ELISA quantification of cytokines in tumor tissues after treatment. Treatment groups: G1, PBS; G2, MCI-NP + Laser; G3, MCI-NP@m; G4, MCI-NP@mPD-1; G5, MCI-NP@mPD-1 + Laser. Statistics: Two-tailed unpaired *t*-test for **i**; log-rank (Mantel-Cox) test for **k**; One-way ANOVA for **l**. Data are presented as mean ± SD. Significance levels: *p < 0.05, **p < 0.01, ***p < 0.001, ****p < 0.0001

PD-1-overexpressing 293FT cells were first generated as a membrane source for subsequent biomimetic camouflage. A lentiviral vector encoding mouse PD-1 fused with an mCherry reporter was constructed and transduced into 293FT cells, yielding a stable PD-1-overexpressing cell line (293FT-PD-1) [26–28]. CLSM analysis revealed strong mCherry fluorescence predominantly localized at the cell periphery, which exhibited substantial colocalization with the plasma membrane dye DiO, confirming successful membrane localization of the PD-1 fusion protein (Fig. 5a). Flow cytometric analysis further demonstrated a high proportion of PD-1-positive cells (> 99%; Fig. 5b), indicating efficient and homogeneous expression across the cell population.

To endow the nanoplatform with PD-L1 immune checkpoint-blocking capability, plasma membranes derived from the engineered 293FT-PD-1 cells (mPD-1) were isolated and coated onto the MCI-NP via membrane extrusion. DLS analysis showed that membrane cloaking resulted in an increase in the hydrodynamic diameter from 98.5 to 108.1 nm, accompanied by a shift in zeta potential from −21.1 to −25.1 mV (Fig. S13), suggesting the successful membrane coverage on the nanoparticle surface. TEM further revealed a uniform and continuous shell surrounding the nanoparticle core in MCI-NP@mPD-1, providing direct morphological evidence of effective membrane coating (Fig. 5c). The retention of membrane-associated proteins after the coating process was further examined at the protein level. Coomassie Brilliant Blue staining showed that the protein band pattern of MCI-NP@mPD-1 closely resembled that of isolated mPD-1 membranes, whereas no detectable protein signals were observed in uncoated MCI-NP, confirming successful preservation of membrane proteins on the nanoparticle surface (Fig. 5d). Consistently, Western blot analysis identified a distinct PD-1 band at approximately 55 kDa in MCI-NP@mPD-1, further validating the presence of PD-1 membrane proteins following the coating process (Fig. 5d). To evaluate the colloidal stability of MCI-NP@mPD-1, nanoparticles were dispersed in medium supplemented with 10% fetal bovine serum, and their hydrodynamic size was monitored by DLS over a 12-day period. As shown in Fig. 5e, MCI-NP@mPD-1 exhibited no appreciable change in particle size throughout the observation period, indicating good stability after membrane coating. Furthermore, the pharmacokinetics of MCI-NP@mPD-1 were evaluated using MSA-2 as a representative analyte for quantification. Plasma analysis revealed a half-life (t_1/2_) of 1.28h and an area under the concentration–time curve (AUC_0-6_) of 1348.33 ng h mL^−1^ following intravenous administration (Fig. S14), indicating their relatively rapid systemic clearance, which may help minimize prolonged systemic exposure and reduce the risk of potential off-target toxicity.

To validate the PD-1/PD-L1-dependent targeting, the cellular uptake and in vivo biodistribution of MCI-NP@mPD-1 were systematically evaluated. In IFN-γ-stimulated Hepa1-6 cells with elevated PD-L1 expression, MCI-NP@mPD-1 exhibited markedly enhanced intracellular fluorescence compared with wild-type cell membrane-coated nanoparticles lacking PD-1 (MCI-NP@m) (Fig. S15). This enhanced uptake was substantially reduced by PD-L1 antibody blockade, confirming a PD-1/PD-L1-mediated cellular recognition mechanism. As shown in Fig. 5f, in vivo fluorescence imaging following intravenous administration revealed that MCI-NP@mPD-1 achieved markedly enhanced and sustained tumor accumulation compared with free ICG, MCI-NP, and MCI-NP@m, with an approximately fivefold increase in tumor-associated fluorescence intensity relative to uncoated MCI-NP (Fig. S15b). Tumor fluorescence intensity reached its maximum at 24 h post-injection, which was therefore selected as the optimal irradiation time point for subsequent intravenous photothermal immunotherapy studies. Together, these results demonstrate that PD-1 membrane camouflage confers effective PD-L1-dependent tumor targeting at both cellular and animal levels.

### In Vivo Photothermal Performance and Antitumor Efficacy of MCI-NP@mPD-1

Prior to efficacy evaluation, the in vivo biocompatibility of MCI-NP@mPD-1 was examined. MCI-NP@mPD-1 exhibited negligible hemolytic activity across a broad concentration range (10–800 μg mL^−1^) and induced no detectable abnormalities in hematological parameters, serum biochemistry, or the histology of major organs following intravenous administration (Fig. S16), indicating a favorable biosafety profile suitable for systemic antitumor application. To assess the in vivo photothermal performance, a Hepa1-6 subcutaneous tumor model was established and treated according to the protocol shown in Fig. 5g. Infrared thermal imaging was used to monitor tumor-site temperature changes upon 808 nm laser irradiation (0.8 W cm^−2^, 10 min) 24h after intravenous administration. As shown in Fig. 5h, i, tumors treated with MCI-NP + Laser reached a moderate temperature of approximately 42.7 °C, whereas the MCI-NP@mPD-1 + Laser group exhibited a pronounced temperature elevation, with peak values reaching 58.1 °C, well above the threshold required for effective photothermal ablation (> 45 °C) [15, 16]. This enhanced photothermal effect is attributed to the improved tumor accumulation and retention of MCI-NP@mPD-1, consistent with the biodistribution results (Fig. 5f), where membrane-coated nanoparticles showed increased tumor localization mediated by PD-1/PD-L1 interactions.

Encouraged by the favorable tumor targeting and photothermal performance, the antitumor efficacy of MCI-NP@mPD-1 was further evaluated in vivo. Tumor growth curves, together with representative tumor-bearing mouse images, revealed pronounced differences among treatment groups (Figs. 5j and S17a). Tumors in the control group (G1) grew rapidly, whereas treatment with MCI-NP + Laser (G2) or MCI-NP@m alone (G3) resulted in only partial growth inhibition. The superior performance of MCI-NP@mPD-1 (G4) over MCI-NP@m (G3) indicates that PD-1/PD-L1 axis blockade conferred by the PD-1-decorated membrane contributes to antitumor efficacy. Notably, MCI-NP@mPD-1 + Laser achieved robust tumor suppression, highlighting the advantage of integrating biomimetic targeting, PTT, and immunomodulation within a single nanoplatform. Survival analysis further confirmed the superior therapeutic outcome of the combined treatment (Fig. 5k). Mice treated with MCI-NP@mPD-1 + Laser exhibited a significant extension of survival, reaching an 80% survival rate at day 60 (*p* < 0.001), markedly outperforming all other groups. Throughout the treatment period, no significant body weight loss was observed among different groups (Fig. S17b), further supporting the in vivo safety of the MCI-NP@mPD-1. To further evaluate the local safety of laser irradiation during photothermal treatment, H&E staining was performed on normal skin tissues surrounding the tumor irradiation area. No detectable histological abnormalities, such as inflammatory infiltration, tissue necrosis, hemorrhage, or structural disruption, were observed in the adjacent skin tissues from the MCI-NP@mPD-1 + Laser-treated mice, suggesting that the photothermal treatment did not cause apparent damage to the surrounding normal tissues under the applied irradiation conditions (Fig. S18).

### MCI-NP@mPD-1-Mediated Remodeling of the Immune Status of the TME

Given the pronounced antitumor efficacy observed with MCI-NP@mPD-1-based photothermal immunotherapy, we next characterized the immunological alterations within the tumor microenvironment. ELISA analysis was performed to quantitatively assess key cytokines within tumor tissues, including IFN-γ, granzyme B (GzmB), IFN-β, TNF-α, TGFβ1, and IL-10. As shown in Fig. 5l, the MCI-NP@mPD-1 + Laser group exhibited a marked elevation of effector and pro-inflammatory cytokines relative to the PBS control, with IFN-γ, GzmB, IFN-β, and TNF-α increased by 8.7-, 2.6-, 3.8-, and 4.9-fold, respectively. These coordinated changes are indicative of enhanced cytotoxic T cell activity together with activation of STING-associated innate immune signaling. In contrast, the immunosuppressive cytokines TGFβ1 and IL-10 were significantly reduced, suggesting an effective alleviation of tumor-associated immune suppression.

**Fig. 6:**
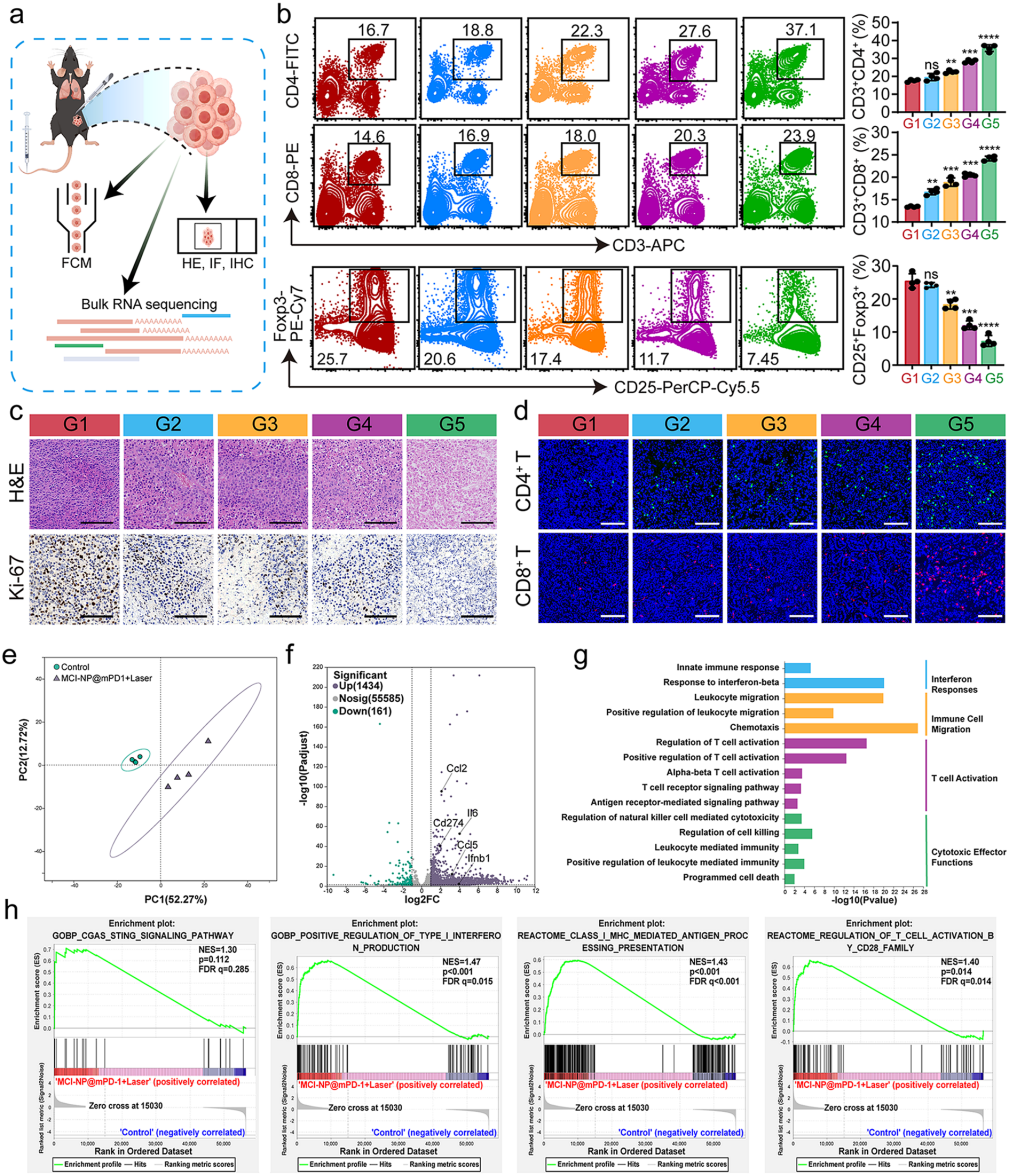
MCI-NP@mPD-1-mediated remodeling of the immune status of TME. Treatment groups: G1, PBS; G2, MCI-NP + Laser; G3, MCI-NP@m; G4, MCI-NP@mPD-1; G5, MCI-NP@mPD-1 + Laser. **a** Schematic illustration of the experimental workflow for TME analysis, including tumor harvest followed by histological staining, immunofluorescence, flow cytometry, and bulk RNA sequencing. **b** Flow cytometric analysis of intratumoral T cell populations following different treatments, showing the proportions of CD3^+^ CD4^+^ T cells, CD3^+^ CD8^+^ T cells, and Tregs (gated within CD4^+^ T cells) within the TME. n = 4; One-way ANOVA; data are presented as mean ± SD; **p < 0.01, ***p < 0.001 ****p < 0.0001. **c** Representative H&E staining and Ki67 immunohistochemical analysis of tumor sections from different treatment groups. **d** Immunofluorescence staining of tumor sections illustrating CD4^+^ and CD8^+^ T cell infiltration following different treatments. **e** PCA of bulk RNA-seq data from tumor tissues collected from PBS and MCI-NP@mPD-1 + Laser groups (n = 4). **f** Volcano plot showing differentially expressed genes between PBS and MCI-NP@mPD-1 + Laser groups, highlighting broad transcriptional changes associated with immune activation. **g** GO enrichment analysis of upregulated genes highlighting selected immune-related biological processes, including interferon responses, immune cell migration, T cell activation, and cytotoxic effector functions. **h** GSEA showing enrichment of immune-related pathways in the MCI-NP@mPD-1 + Laser group

To further delineate the cellular basis underlying tumor immune microenvironment remodeling, tumor tissues were harvested after treatment and subjected to integrated histological, immunofluorescence, flow cytometric, and transcriptomic analyses (Fig. 6a). Flow cytometric analysis revealed a pronounced reshaping of the intratumoral T cell landscape in the MCI-NP@mPD-1 + Laser group, characterized by enhanced effector T cell infiltration together with attenuation of immunosuppressive Tregs. As shown in Figs. 6b, S19, and S20, in the MCI-NP@mPD-1 + Laser group, CD3^+^ CD4^+^ and CD3^+^ CD8^+^ T cells accounted for 35.8% and 24.2%, respectively, compared with 17.6% and 13.4% in the PBS group; whereas the proportion of Tregs was reduced to 7.3% of CD4^+^ T cells from 25.5% in the PBS group. Consistent with these findings, histological and immunofluorescence analyses corroborated treatment-induced remodeling of the TME at the tissue level. H&E staining revealed more pronounced nuclear condensation and cellular damage in tumors treated with MCI-NP@mPD-1 + Laser, accompanied by a marked reduction in Ki67-positive cells (Fig. 6c), indicating effective tumor ablation and suppressed proliferation. Immunofluorescence staining further demonstrated substantially increased CD4^+^ and CD8^+^ T cell signals in tumor sections from the combination group (Fig. 6d), confirming enhanced effector T cell infiltration.

To further characterize treatment-induced transcriptional reprogramming of the TME, bulk RNA sequencing was performed on tumor tissues collected from the PBS and MCI-NP@mPD-1 + Laser groups (n = 4). Principal component analysis (PCA) revealed a clear separation between the two groups with no cluster overlap, indicating a pronounced treatment-driven transcriptional shift in the TME (Fig. 6e). Differential gene expression (DEG) analysis further demonstrated extensive transcriptional remodeling following combination therapy, with upregulated genes broadly associated with inflammatory chemokine signaling and type I interferon responses, consistent with enhanced immune activation and STING-associated innate immune signaling (Fig. 6f). Gene Ontology (GO) enrichment analysis of upregulated genes demonstrated significant enrichment of immune-related biological processes, particularly type I interferon responses, immune cell migration, and T cell activation, supporting enhanced intratumoral T cell infiltration and adaptive antitumor immune activation (Fig. 6g). To further interrogate coordinated pathway-level alterations, gene set enrichment analysis (GSEA) was performed (Fig. 6h). Immune-related pathways, including type I interferon signaling, MHC class I antigen processing and presentation, and CD28 family-mediated T cell activation, were significantly enriched in the MCI-NP@mPD-1 + Laser group. Notably, the cGAS-STING signaling pathway also showed enrichment, consistent with the enhanced IFN-β production and STING-associated innate immune activation observed at the cytokine and cellular levels (Fig. 3g–i). Collectively, these transcriptomic analyses demonstrate that MCI-NP@mPD-1-based photothermal immunotherapy elicits coordinated immune activation, effectively converting the tumor microenvironment into a more immune-responsive state.

### MCI-NP@mPD-1-Mediated Inhibition of Lung Metastasis and Immune Memory Induction

**Fig. 7:**
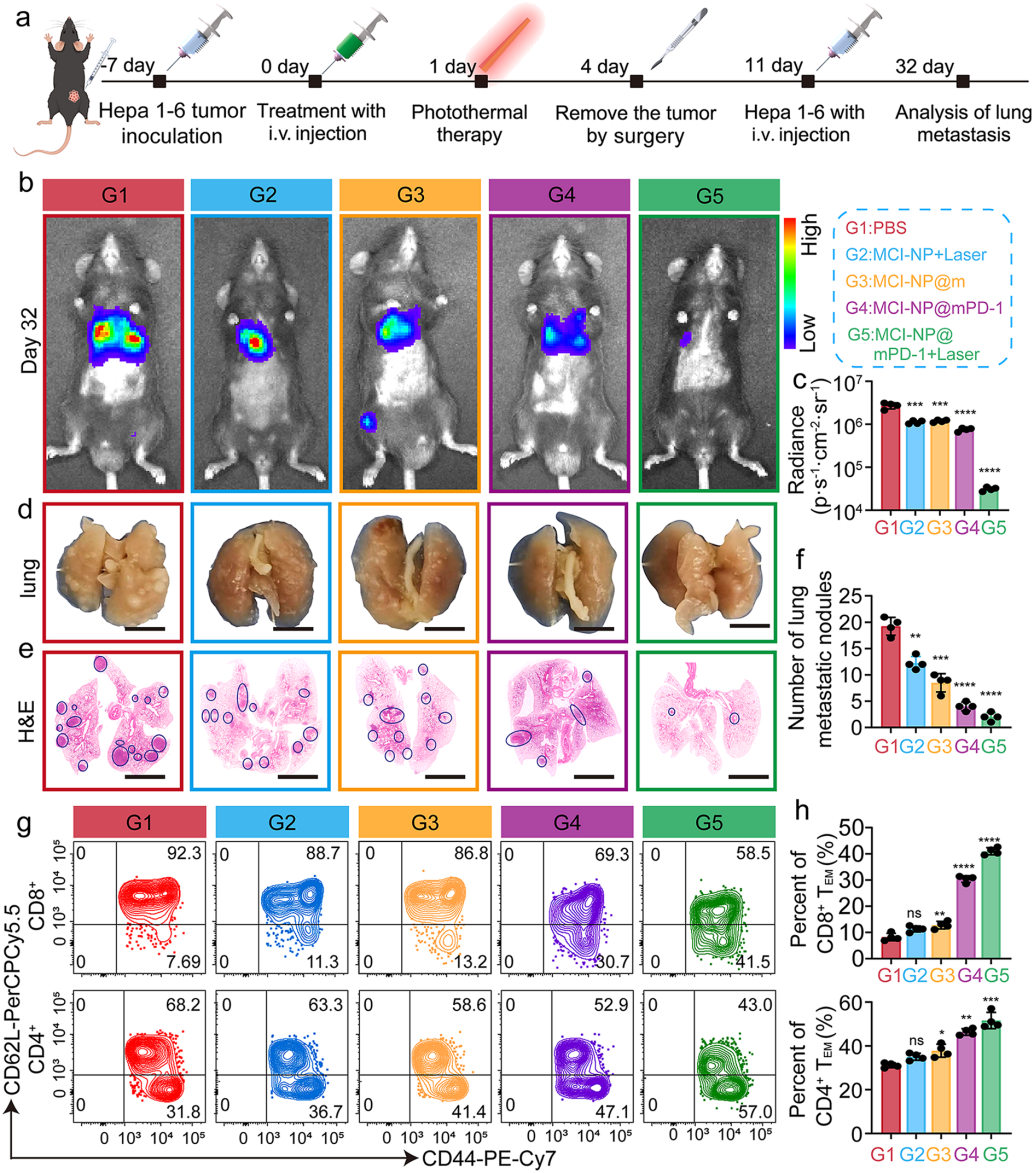
MCI-NP@mPD-1-mediated suppression of pulmonary metastasis and induction of immune memory. Treatment groups: G1, PBS; G2, MCI-NP + Laser; G3, MCI-NP@m; G4, MCI-NP@mPD-1; G5, MCI-NP@mPD-1 + Laser. **a** Schematic illustration of the experimental design for the postoperative pulmonary metastasis model (n = 4). **b** Representative in vivo bioluminescence images of mice bearing pulmonary metastases following intravenous challenge with Hepa1-6-Luc cells after different treatments. **c** Quantification of pulmonary bioluminescence signals corresponding to **b**. **d** Representative photographs of excised lungs from different treatment groups, showing the macroscopic metastatic burden. **e** Representative H&E staining of lung sections from different groups, revealing metastatic tumor nodules. **f** Quantification of metastatic nodules in the lungs across different treatment groups. **g** Representative flow cytometric density plots of effector memory T cells (CD44^+^ CD62L^−^) in peripheral blood gated on CD8^+^ CD44^+^ or CD4^+^ CD44^+^ T cells following different treatments. **h** Statistical analysis of CD8^+^ and CD4^+^ effector memory T cell (T_EM_) populations within the CD44.^+^ T cell subsets corresponding to **g**. Statistics: One-way ANOVA for **c, f,** and** h**, compared with the PBS control group. Data are presented as mean ± SD. Significance levels: *p < 0.05, **p < 0.01, ***p < 0.001, ****p < 0.0001

Tumor metastasis, driven by the dissemination of tumor cells from primary lesions and subsequent colonization of distant organs, is a critical determinant of malignant progression. Given the robust antitumor efficacy of MCI-NP@mPD-1-based photothermal immunotherapy in primary tumors, we next examined whether this treatment could elicit durable antitumor immune memory capable of preventing postoperative tumor metastasis. A postoperative pulmonary metastasis model was established as illustrated in Fig. 7a. When tumors reached 50–80 mm^3^, tumor-bearing mice were randomized into five groups: PBS (G1), MCI-NP + Laser (G2), MCI-NP@m (G3), MCI-NP@mPD-1 (G4), and MCI-NP@mPD-1 + Laser (G5). Following intravenous administration, laser irradiation (808 nm, 0.8 W cm^−2^, 10 min) was applied 24h later. Residual primary tumors were surgically resected on day 4, and Hepa1-6-Luc cells were intravenously injected on day 11 to establish lung metastases. Tumor dissemination was monitored by bioluminescence imaging three weeks after tumor cell challenge (Fig. 7b). Mice in the PBS group exhibited strong pulmonary bioluminescence signals, indicative of extensive lung metastasis. In contrast, mice treated with MCI-NP + Laser, MCI-NP@m, or MCI-NP@mPD-1 displayed reduced but still detectable lung signals, suggesting limited antimetastatic protection. Notably, the MCI-NP@mPD-1 + Laser group showed minimal pulmonary bioluminescence, indicating effective suppression of tumor metastasis (Fig. 7c). Consistent with the in vivo imaging results, gross examination of excised lungs revealed a severe metastatic burden in the PBS group, whereas fewer metastatic nodules were observed in mice treated with MCI-NP + Laser, MCI-NP@m, or MCI-NP@mPD-1 (Fig. 7d). Strikingly, lungs from the MCI-NP@mPD-1 + Laser group exhibited only sparse metastatic lesions. These findings were further confirmed by histological analysis, with H&E staining showing the highest number of metastatic nodules in the PBS group and the lowest in the MCI-NP@mPD-1 + Laser group (Fig. 7e, f). To determine whether metastasis suppression was associated with the induction of immune memory, peripheral blood leukocytes were isolated and analyzed by flow cytometry. As shown in Figs. 7g, h and S21, mice treated with MCI-NP@mPD-1 + Laser exhibited markedly increased proportions of effector memory T cells (CD44^+^ CD62L^−^ cells), with T_EM_ accounting for 41.0% of CD8^+^ CD44^+^ T cells and 51.6% of CD4^+^ CD44^+^ T cells, respectively. Collectively, these results demonstrate that MCI-NP@mPD-1-based photothermal immunotherapy not only suppresses primary HCC but also induces systemic antitumor immune memory, providing sustained protection against tumor dissemination.

As an additional evaluation of the therapeutic efficacy of MCI-NP@mPD-1-based treatment, a bilateral Hepa1-6 model was established, in which only the primary tumor received laser irradiation after intravenous administration. Compared with PBS, MCI-NP@mPD-1 + Laser markedly inhibited the growth of both irradiated primary tumors and non-irradiated contralateral tumors (Fig. S22). Given the systemic administration route, this effect may result from both nanoparticle-mediated tumor delivery and treatment-induced antitumor immune responses, further supporting the systemic therapeutic potential of MCI-NP@mPD-1-based photothermal-immunotherapy.

Recent studies have increasingly explored PTT-STING combination strategies, including MSA-2/Mn^2+^-loaded mesoporous polydopamine platforms, NIR-II phototheranostic systems for cGAS-STING activation, thermoresponsive exosome-liposome hybrids, and implantable depots for sequential immunomodulation [6, 10, 12, 38]. These approaches support the concept that photothermal damage can enhance tumor immunogenicity, while STING activation amplifies innate immune signaling. Compared with these systems, MCI-NP@mPD-1 integrates carrier-free Cu^2+^-coordinated co-assembly of ICG and MSA-2 to couple photothermal ablation with STING activation, while PD-1 membrane camouflage simultaneously enhances tumor accumulation and provides localized PD-1/PD-L1 blockade. Together with STING-KO validation, transcriptomic evidence of immune reprogramming, and the suppression of postoperative metastasis and bilateral tumors, these findings support the development of an HCC-oriented photothermal-immunotherapeutic platform capable of addressing both immune activation and adaptive immune resistance. Thus, beyond the integration of photothermal therapy and STING activation, MCI-NP@mPD-1 provides a strategy for simultaneously amplifying antitumor immunity and overcoming treatment-induced adaptive immune resistance in immune-cold HCC.

## Conclusion

Given the pivotal role of the STING pathway in sensitizing immunologically cold HCC to immune responsiveness, we developed a multifunctional, carrier-free nanomedicine (MCI-NP) co-assembled from the STING agonist MSA-2 and the photothermal agent ICG via Cu^2+^-mediated coordination, enabling efficient photothermal ablation and robust STING-driven innate immune activation. MCI-NP exhibited enhanced photothermal conversion efficiency, effectively inducing immunogenic cell death (ICD) under near-infrared irradiation while simultaneously activating the STING pathway in antigen-presenting cells. In a syngeneic Hepa1-6 HCC model, intratumoral administration of MCI-NP elicited pronounced antitumor responses, although late-stage tumor regrowth was observed and was accompanied by upregulated PD-L1 expression. To overcome this adaptive immune escape, MCI-NP was cloaked with PD-1-overexpressing engineered cell membranes to generate MCI-NP@mPD-1, thereby enhancing tumor accumulation and alleviating PD-1/PD-L1-mediated immunosuppression. A single treatment with MCI-NP@mPD-1 achieved near-complete tumor regression, reshaped the immune landscape toward an immune-active state, promoted effector T cell infiltration, and induced durable systemic immune memory that protected against postoperative pulmonary metastasis. Overall, this work highlights the potential of integrating photothermal ablation, innate immune stimulation, and biomimetic checkpoint blockade to overcome immune escape and extend the benefits of localized tumor therapy to long-term systemic immune protection.

## Supplementary Information

Below is the link to the electronic supplementary material.

Supplementary file1 (DOCX 6539 KB)
