# Supplementary material for: Biomimetic PD-1-Functionalized Immunostimulatory Nanomedicine Enables STING Activation and Durable Antitumor Immunity in Hepatocellular Carcinoma
Source: Nanomicro Lett. 2026 Jul 30;19:9. doi: 10.1007/s40820-026-02320-7 (PMC13424038; doi:10.1007/s40820-026-02320-7)
Supplement: Supplementary file 1 — Supplementary file1 (DOCX 6539 KB) [file 40820_2026_2320_MOESM1_ESM.docx]

Supporting Information for

**Biomimetic PD-1-Functionalized Immunostimulatory Nanomedicine Enables STING Activation and Durable Antitumor Immunity in Hepatocellular Carcinoma**

Yanbing Cao^1,3#^, Xinyi Lin^1,3#^*, Bingchen Wu^1,3#^, Yang Li^5^, Wenrui Zhu^1,3^, Binxin Liu^1,3^, Aixian Zheng^1,3^, Lingjie Wu^1,3^, Yanyang Wang^2,4^*, Xiaolong Liu^1,3^*, Ming Wu^1,3^*

^1^ The United Innovation of Mengchao Hepatobiliary Technology Key Laboratory of Fujian Province, Mengchao Hepatobiliary Hospital of Fujian Medical University, Fuzhou 350025, P. R. China

^2^ Department of Radiation Oncology, General Hospital of Ningxia Medical University, Yinchuan 750004, P. R. China

^3^ The Liver Center of Fujian Province, Fujian Medical University, Fuzhou 350025, P. R. China

^4^ Institute of Medical Sciences, General Hospital of Ningxia Medical University, Yinchuan 750004, P. R. China

^5^ State Key Laboratory of Structural Chemistry, Fujian Institute of Research on the Structure of Matter, Chinese Academy of Sciences, Fuzhou, P. R. China

^#^Yanbing Cao, Xinyi Lin, and Bingchen Wu have equal contribution to this paper.

*Corresponding authors. E-mail: [lxy.1210@163.com](mailto:lxy.1210@163.com) (Xinyi Lin ); [wangyy@nxmu.edu.cn](mailto:wangyy@nxmu.edu.cn) (Yanyang Wang ); [xiaoloong.liu@gmail.com](mailto:xiaoloong.liu@gmail.com) (Xiaolong Liu ); [wmmj0419@163.com](mailto:wmmj0419@163.com) (Ming Wu)

**S1 Experimental Section**

**S1.1 Fabrication and characterization**

*Preparation of MCI-NP*: MCI-NP was prepared via a coordination-driven self-assembly strategy. Briefly, 10 mg of MSA-2 (Titan Scientific, Shanghai, China) was dissolved in dimethyl sulfoxide and diluted with ethanol, followed by dropwise addition into an ethanolic solution of CuCl_2_ (1 mL, 10 mg·mL^-1^; Sinopharm Chemical Reagent, Shanghai, China) under continuous stirring. After coordination assembly for 8 h, 1 mL of an ethanol solution of ICG (20 mg·mL^-1^; Titan Scientific, Shanghai, China) was introduced and allowed to further assemble for an additional 2 h. The resulting mixture was slowly injected into ultrapure water to induce nanoparticle formation via solvent exchange, and ethanol was removed by overnight stirring. The nanoparticles were collected by ultracentrifugation (100 000 g, 30 min) and redispersed in ultrapure water to obtain a stable MCI-NP suspension.

*Preparation of PD-1-overexpressing cell membranes (mPD-1)*: PD-1-overexpressing 293FT cells were generated via lentiviral transduction using a PD-1-mCherry fusion construct under the control of a CMV promoter, following procedures adapted from our previously reported membrane engineering strategy [S1-S3]. Stable cell lines were obtained by puromycin selection and monoclonal expansion. Membrane localization and homogeneous expression of PD-1 were confirmed by CLSM and flow cytometry. Plasma membranes were isolated from PD-1-overexpressing 293FT cells via hypotonic lysis and subsequent differential centrifugation. The resulting membrane fractions were collected by ultracentrifugation, resuspended in Tris-based buffer, and stored at −80 °C until use. Protein concentrations were quantified by BCA assay. Wild-type 293FT cells served as the source for control cell membranes, which were isolated and stored following the same protocols as described for mPD-1.

*Preparation of MCI-NP@mPD-1*: MCI-NP@mPD-1 was prepared by coating MCI-NP with mPD-1 via a membrane fusion-extrusion strategy. Briefly, mPD-1 were mixed with MCI-NP at a mass ratio of 1:1 in PBS (pH 7.4), followed by probe sonication in an ice bath to promote membrane dispersion. The mixture was then incubated at 37 °C under gentle agitation to facilitate membrane-nanoparticle coating. The resulting suspension was sequentially extruded through polycarbonate membranes with decreasing pore sizes to obtain uniformly membrane-coated MCI-NP@mPD-1. MCI-NP@m was synthesized using the same procedures as described above, but employing wild-type membranes as the coating source.

*Physicochemical Characterization*: The morphology and structure of both MCI-NP and MCI-NP@mPD-1 were characterized by TEM, and elemental distribution of MCI-NP was analyzed using HAADF-STEM elemental mapping (TALOS F2000, FEI). FT-IR spectra were recorded using an FT-IR spectrometer (Spectrum-2000, PerkinElmer). Optical absorption spectra were obtained using a UV-Vis spectrophotometer (SpectraMax M5, Molecular Devices). Hydrodynamic diameter and zeta potential were measured by DLS (Nano ZS, Malvern Instruments). Surface elemental composition and chemical states were analyzed by XPS (K-Alpha+, Thermo Scientific).

The loading content of MSA-2 and ICG in MCI-NP was quantified by high-performance liquid chromatography (HPLC; Agilent 1260 Infinity). The pH-dependent drug release behavior was evaluated using a dialysis method. Briefly, MCI-NP was dispersed in phosphate-buffered saline (PBS, pH 7.4) or acidic buffer (pH 5.5) and incubated at 37 °C with gentle shaking. At predetermined time points, aliquots of the external buffer were collected for HPLC analysis and replaced with fresh buffer. The cumulative release profiles of MSA-2 and ICG were calculated accordingly. Retention of membrane proteins after coating was examined by Coomassie Brilliant Blue staining and Western blot analysis, with PD-1 used as a representative membrane marker.

Photostability and photothermal stability of MCI-NP were systematically evaluated in comparison with free ICG and a physical mixture of Cu^2+^, MSA-2, and ICG. For photostability analysis, free ICG, MCI-NP, and the physical mixture (Cu^2+^+MSA-2+ICG) were dispersed in deionized water at ICG concentration of 100 μg·mL^-1^. All samples were exposed to continuous 808 nm laser irradiation (0.8 W·cm^-2^). At predetermined time points (2, 4, and 6 min), UV-Vis-NIR absorption spectra were immediately recorded to monitor irradiation-induced changes in optical absorbance and evaluate the photostability of each formulation. For photothermal stability evaluation, free ICG, MCI-NP, and the physical mixture (100 μg·mL^-1^ for each group) were subjected to multiple cycles of 808 nm laser irradiation (0.8 W·cm^-2^, on/off cycles). Temperature changes during each heating-cooling cycle were recorded using an infrared thermal imaging system to assess the reproducibility and stability of photothermal conversion performance.

***S1.2 In vitro* studies**

*Cell culture and BMDC preparation*: Mouse hepatocellular carcinoma Hepa1-6 cells and Hepa1-6-Luc cells, normal mouse hepatocytes (BNL CL.2), and 293FT cells were cultured in Dulbecco’s modified Eagle medium (DMEM) supplemented with 10% fetal bovine serum (FBS) and 1% penicillin-streptomycin under standard conditions (37 °C, 5% CO_2_). Bone marrow-derived dendritic cells (BMDCs) were generated from murine bone marrow progenitors according to established protocols and used at an immature stage unless otherwise specified.

*Cellular uptake*: Cellular uptake of different formulations was evaluated by confocal laser scanning microscopy (CLSM; Zeiss LSM780) based on the intrinsic fluorescence of ICG. Hepa1-6 cells were incubated with MCI-NP or membrane-coated formulations at a final concentration of 50 μg·mL^-1^ for 2, 4, or 8 h, followed by fixation and nuclear counterstaining prior to imaging. To evaluate PD-1/PD-L1-dependent uptake, Hepa1-6 cells were pretreated with IFN-γ (20 ng·mL^-1^) for 24 h to induce PD-L1 expression and subsequently incubated with MCI-NP@mPD-1 or control nanoparticles for 4 h. For blocking experiments, cells were preincubated with a PD-L1-neutralizing antibody (BioXCell, BE0383) for 1 h prior to nanoparticle exposure. To further evaluate the uptake of MCI-NP by antigen-presenting cells, BMDCs were seeded in 6-well plates at a density of 1 × 10^6^ cells per well and incubated with MCI-NP-containing medium for 2, 4, or 6 h. After incubation, cells were washed three times with PBS to remove free nanoparticles and collected for flow cytometric analysis based on the intrinsic fluorescence of ICG.

*In vitro cytotoxicity and photothermal killing*: Cell viability was assessed using Cell Counting Kit-8 (CCK-8, Dojindo Laboratories, Kumamoto, Japan). Hepa1-6 and BNL CL.2 cells were incubated with MCI-NP at concentrations ranging from 0 to 400 μg·mL^-1^ for 48 h, followed by viability assessment according to the manufacturer’s instructions. For photothermal cytotoxicity, Hepa1-6 cells were incubated with MCI-NP at the indicated concentrations for 24 h, irradiated with an 808 nm laser (0.8 W·cm^-2^, 10 min), and further cultured for 24 h before viability analysis. Live/dead staining (Invitrogen, Eugene, OR, USA) and Annexin V/PI apoptosis assays (Dojindo Laboratories, Kumamoto, Japan) were additionally performed to visualize and quantify photothermal-induced cell death.

*Evaluation of immunogenic cell death*: Immunogenic cell death (ICD) was assessed by analyzing calreticulin (CRT) exposure, high-mobility group box 1 (HMGB1) translocation, and extracellular ATP release. Hepa1-6 cells were treated with MCI-NP (60 μg·mL^-1^) with or without laser irradiation (808 nm, 0.8 W·cm^-2^, 10 min), followed by 24 h incubation. CRT (Abcam, ab92516) exposure and HMGB-1 (Abcam, ab18256) subcellular localization were examined by immunofluorescence staining and CLSM. For ATP analysis, culture supernatants were collected 4 h after treatment, cleared by centrifugation, and analyzed using a luciferase-based ATP assay according to the manufacturer’s protocol (Beyotime, S0026).

*BMDC maturation and STING pathway activation*: To evaluate BMDC maturation, culture supernatants from treated Hepa1-6 cells were collected 24 h after treatment and mixed with fresh medium at a 1:1 ratio to generate conditioned media. Immature BMDCs were incubated with conditioned media for 24 h, and maturation was analyzed by flow cytometry based on CD80 (eBioscience™, 12-0801-82) and CD86 (eBioscience™, 25-0862-82) expression within the CD11c^+^ (eBioscience™, 17-0114-82) population. Cytokine secretion, including TNF-α (Boster, EK0527) and IL-6 (Boster, EK0411), was quantified by ELISA.

For direct assessment of STING signaling, BMDCs were incubated with MCI-NP at concentrations of 0, 20, and 40 μg·mL^-1^ for 48 h. IFN-β (FANKEW, F2124-A) and IL-6 (Boster, EK0411) levels in the culture supernatants were quantified by ELISA. Activation of the STING pathway was further examined by Western blot analysis of total and phosphorylated STING (CST, 13647; CST, 72971), TBK1 (CST, 3504; CST 5483), and IRF3 (CST, 4302; CST 4947).

*Analysis of PD-L1 expression induced by IFN-γ:* Hepa1-6 cells were seeded at a density of 3 × 10^5^ cells per well in 6-well plates (for flow cytometry) or glass-bottom culture dishes (for immunofluorescence imaging). After 24 h, cells were treated with recombinant mouse IFN-γ at final concentrations of 0, 10, or 20 ng·mL^-1^ for 48 h. For flow cytometric analysis, cells were harvested, blocked with 5% BSA, and incubated with anti-mouse PD-L1-Alexa Fluor 488 antibody (ab252436, Abcam) for 60 min at room temperature in the dark. After washing and resuspension, PD-L1 expression was analyzed by flow cytometry. For immunofluorescence analysis, cells cultured in glass-bottom dishes were fixed with 4% paraformaldehyde, blocked with 5% BSA, and incubated with anti-mouse PD-L1-Alexa Fluor 488 antibody for 2 h at room temperature. Nuclei were counterstained with DAPI, and fluorescence images were acquired using a confocal laser scanning microscope.

***S1.3 In vivo* studies**

*Animal models*: Male C57BL/6 mice (6-8 weeks old) were purchased from Beijing Vital River Laboratory Animal Technology Co., Ltd. STING-knockout (STING-KO) C57BL/6 mice were obtained from Cyagen Biosciences (Guangzhou, China). Subcutaneous tumor models were established by subcutaneous injection of 3 × 10^6^ Hepa1-6 cells suspended in 50% Matrigel (Corning, 356234) /PBS (100 μL per mouse) into the right flank of C57BL/6 mice. Bilateral Hepa1-6 tumor models were established by inoculating Hepa1-6 cells into both flanks of C57BL/6 mice (right flank: 1 × 10^6^ cells; left flank: 5 × 10^5^ cells). When primary tumors reached approximately 100 mm^3^, mice received intravenous administration of MCI-NP@mPD-1 followed by laser irradiation applied only to the primary tumor 24 h later. Tumor growth at both primary and contralateral sites was monitored throughout the study. Tumor volume was monitored using digital calipers and calculated as V = L × W^2^ × 0.5. Treatments were initiated when tumor volumes reached approximately 100 ± 10 mm^3^. Animals exhibiting persistent hunching posture, markedly reduced activity, severe weight loss, or other predefined humane endpoint criteria were euthanized according to institutional animal care guidelines.

*In vivo biodistribution*: For intratumoral diffusion assays, MCI-NP (50 μL, equivalent to 20 μg MSA-2) was locally injected into established Hepa1-6 tumors (~150 mm^3^). Near-infrared fluorescence imaging was performed at 0.5, 1, 2, 4, 8, 12, 24, and 36 h post-injection using an NIR imaging system (UNITED WELL NW-1000). For systemic biodistribution, tumor-bearing mice received intravenous injection of MCI-NP@mPD-1 (5 mg·kg^-1^, 100 μL). Whole-body NIR-II fluorescence imaging was conducted at predetermined time points (0.5-36 h). Major organs and tumors were harvested after the final imaging for ex vivo fluorescence analysis.

*In vivo pharmacokinetic study*: C57BL/6 mice received a single intravenous injection of MCI-NP@mPD-1 at a dose of 5 mg·kg^-1^ in a volume of 100 μL. Blood samples were collected from the retro-orbital venous plexus into EDTA-anticoagulated tubes at 5, 15, and 30 min and at 1, 2, 4, 6, 12, and 24 h after administration. Samples were centrifuged at 1,500 × g for 5 min at room temperature to obtain plasma, which was stored at -20 °C until analysis. MSA-2 was selected as the representative analyte for pharmacokinetic evaluation. Plasma concentrations of MSA-2 were quantified using a UPLC-MS/MS system consisting of a Shimadzu UPLC 30AD liquid chromatography system (Tokyo, Japan) coupled to an AB Sciex API 5500 triple quadrupole mass spectrometer equipped with an electrospray ionization (ESI) source (Los Angeles, CA, USA). Chromatographic separation was performed on a Shim-pack XR-ODS III C18 column (2.0 × 50 mm, 1.6 μm) equipped with a Shim-pack GIST-HP (G) guard column (2.1 × 10 mm, 1.6 μm; Tokyo, Japan). The flow rate was maintained at 0.3 mL·min^-1^. The mobile phase consisted of solvent A (water containing 2 mM ammonium acetate and 0.1% formic acid) and solvent B (acetonitrile containing 0.1% formic acid). The gradient elution program was as follows: 30% B from 0 to 0.1 min, 30-90% B from 0.1 to 1.5 min, 90% B from 1.5 to 2.5 min, 90-30% B from 2.5 to 3.0 min, and 30% B from 3.0 to 3.5 min. MSA-2 was detected in negative electrospray ionization mode by monitoring the deprotonated molecular ion. The mass spectrometric parameters were set as follows: curtain gas, 30 psi; collision gas, 9 psi; ion source gas 1, 55 psi; ion source gas 2, 50 psi; ion spray voltage, -4500 V; and source temperature, 400 °C. Pharmacokinetic parameters were calculated by non-compartmental analysis using Phoenix WinNonlin 8.2 software (Pharsight Corporation, Mountain View, CA, USA). The area under the plasma concentration–time curve from 0 to 6 h (AUC_0-6_) was calculated using the linear trapezoidal method. The terminal elimination half-life (t_1/2_) was calculated as 0.693/k, where k represents the first-order elimination rate constant.

*In vivo photothermal therapy*: Photothermal treatment was performed using an 808 nm near-infrared laser (0.8 W·cm^-2^, 10 min, spot diameter 1 cm). Laser irradiation (MDL-III-808, Changchun New Industries Optoelectronics Technology Co., Ltd.) was applied 24 h after nanoparticle administration. Tumor surface temperatures were continuously monitored using an infrared thermal imaging camera (Ti25, Fluke) to ensure effective photothermal heating.

*In vivo antitumor efficacy*: Tumor-bearing mice were randomly assigned to different treatment groups (n = 5). For intratumoral therapy studies, nanoparticles were administered via intratumoral injection at a dose of 2.5 mg·kg^-1^ in 50 μL PBS. For systemic therapy studies, formulations were administered intravenously via the tail vein at a dose of 5 mg·kg^-1^ in 100 μL PBS. Tumor volumes and body weights were recorded every two days. Mice were euthanized when tumor volume exceeded 1500 mm^3^ or body weight loss exceeded 20%, in accordance with ethical guidelines. For survival analysis, mice were monitored for up to 60 days post-treatment, and survival curves were analyzed using the Kaplan-Meier method with log-rank testing.

*In vivo immune activation and tumor microenvironment analysis*: Tumors were harvested 2 days after treatment for histological analysis and 3 days after treatment for flow cytometric and cytokine analyses. Tumor tissues were processed for H&E staining, Ki67 immunohistochemistry (CST, 9449S), and TUNEL staining (Beyotime, Jiangsu, China) to assess tissue damage and proliferation. Immunofluorescence staining was further performed to evaluate intratumoral CD4 (GB11064) and CD8 (Servicebio, GB11068) T-cell infiltration. For flow cytometric analysis, tumors were enzymatically dissociated into single-cell suspensions and tumor-infiltrating lymphocytes were analyzed for CD3^+^ T cell (eBioscience™, 12-0081-82), CD4^+^ T cell (eBioscience™, 11-0042-85), CD8^+^ T cell (eBioscience™, 12-0081-82), and CD4^+^ Foxp3^+^ (eBioscience™, 25-5773-80) Treg populations. Cytokine levels, including TNF-α (Boster, EK0527), IFN-γ (Boster, EK0375), IFN-β (FANKEW, F2124-A), IL-6 (Boster, EK0411), IL-10 (Boster, EK0417), TGFβ (Boster, EK0515) and granzyme B (Boster, EK1115), in tumor lysates were quantified by ELISA. Selected tumor samples were further subjected to bulk RNA sequencing for transcriptomic analysis.

*Evaluation of STING dependence in vivo*: To assess STING dependency, Hepa1-6 tumors were established in STING-KO mice. Mice received intratumoral injection of MCI-NP or PBS following the same treatment schedule as wild-type mice. Tumor growth, body weight, and final tumor weights were recorded to evaluate therapeutic efficacy in the absence of functional STING signaling.

*Postoperative lung metastasis and immune memory evaluation*: To evaluate antimetastatic immune memory, primary tumors were surgically resected after treatment. Seven days post-surgery, mice were intravenously challenged with Hepa1-6-Luc cells (5 × 10^5^ cells per mouse). Lung metastasis was monitored by bioluminescence imaging at weekly intervals following intraperitoneal injection of D-luciferin (150 mg·kg^-1^). Three weeks after tumor cell challenge, lungs were collected for gross examination and H&E staining to quantify metastatic nodules. Peripheral blood mononuclear cells were isolated and analyzed by flow cytometry to assess effector memory T-cell populations. Effector memory T cells were defined as CD44^+^ (eBioscience™, 25-0441-82) CD62L^−^ (eBioscience™, 45-0621-82) subsets within CD4^+^ and CD8^+^ T cells.

*In vivo biosafety evaluation*: The *in vivo* biosafety of MCI-NP@mPD-1 was assessed by hemolysis assay, hematological and serum biochemical analysis, and histological examination. Fresh mouse red blood cells (RBCs) were isolated by centrifugation and washed with PBS to prepare a 5% RBC suspension. MCI-NP@mPD-1 at different concentrations (0-800 μg·mL^-1^) was incubated with RBCs at 37 °C for 2 h, with PBS and ultrapure water serving as negative and positive controls, respectively. After centrifugation, absorbance of the supernatant was measured at 540 nm, and hemolysis ratios were calculated according to ASTM F756 standards. For systemic safety evaluation, healthy C57BL/6 mice were intravenously injected with MCI-NP@mPD-1 at the therapeutic dose, followed by hematological and serum biochemical analyses. Major organs (heart, liver, spleen, lung, and kidney) were harvested for H&E staining. To further evaluate local tissue safety under photothermal treatment, skin tissues located approximately 2 mm surrounding the laser irradiation site (anterior, posterior, left, and right directions) were collected after treatment and subjected to H&E staining for histological analysis of potential thermal damage.

**S1.4 Statistical Analysis**

All statistical analyses were performed using GraphPad Prism 8.0.5 (GraphPad Software). Data are presented as mean ± standard deviation (SD) or as individual data points with the mean indicated, as specified in the figure legends. Statistical significance between two groups was assessed using two-tailed unpaired Student’s *t*-test, while comparisons among multiple groups were performed using one-way ANOVA as indicated. Survival data were analyzed using the log-rank (Mantel-Cox) test. A *p* value < 0.05 was considered statistically significant (**p* < 0.05, ***p* < 0.01, ****p* < 0.001, *****p* < 0.0001).

**S2 Supplementary Data**


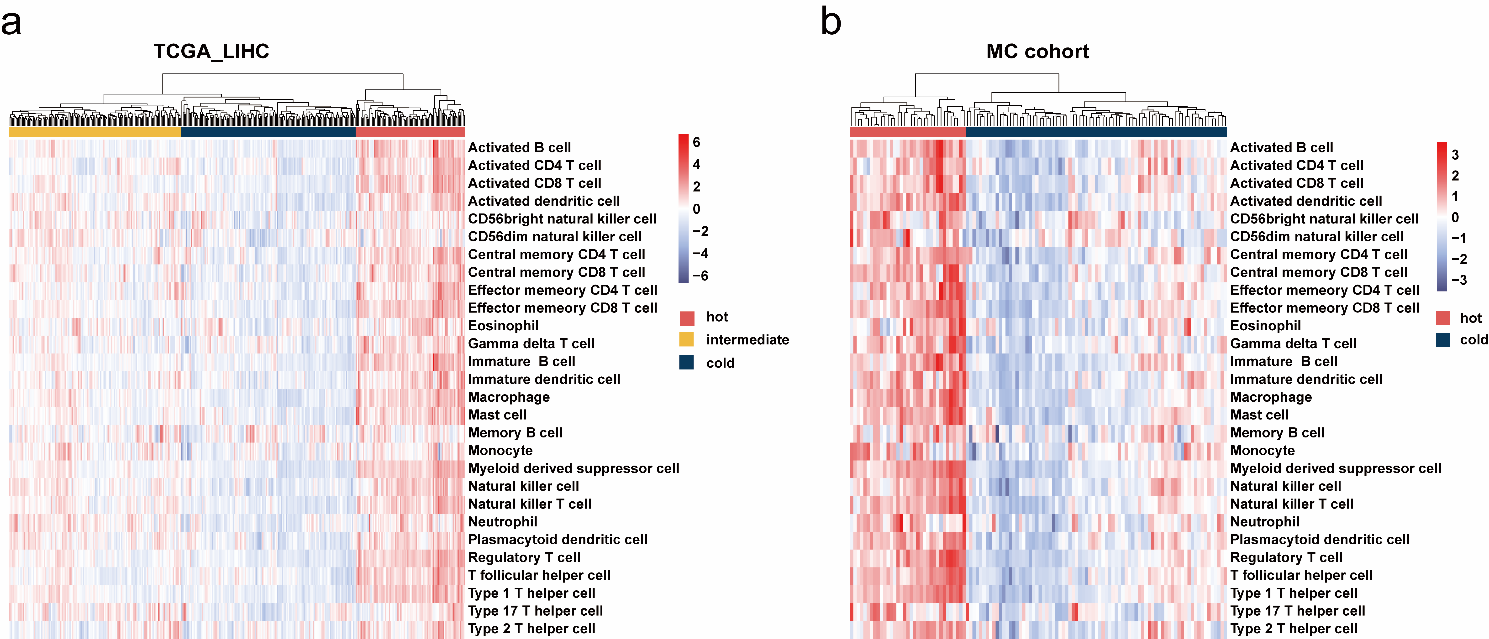


**Fig. S1** Immune classification of HCC samples based on immune cell signature expression in **a** the TCGA-LIHC cohort and **b** the MC cohort.


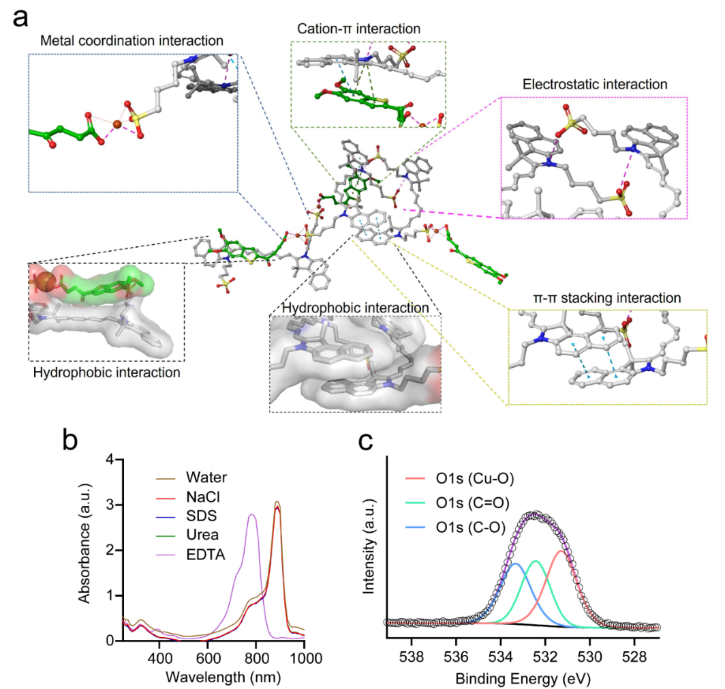


**Fig. S2** Mechanistic insights and characterization of MCI-NP self-assembly. **a** Predicted binding configuration of Cu^2+^, ICG and MSA-2 derived from molecular docking. **b** UV-vis absorption spectra of MCI-NP following treatment with SDS, EDTA, NaCl, or urea to probe intermolecular interactions. **c** High-resolution O 1s XPS spectra of MCI-NP, highlighting Cu-O coordination features.


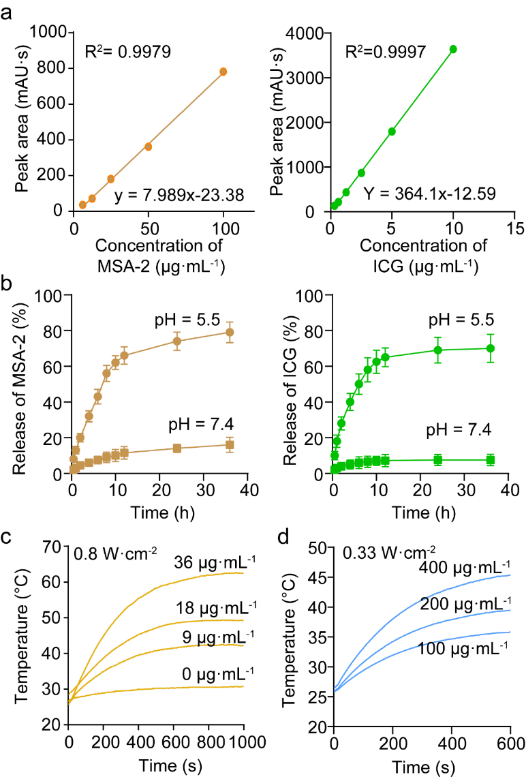


**Fig. S3** Supplementary characterization of drug loading, pH-responsive release, and photothermal performance of MCI-NP. **a** HPLC calibration curve of MSA-2 and ICG. **b** In vitro cumulative release profile of MSA-2 and ICG from MCI-NP at pH 5.5 and pH 7.4. **c** Temperature elevation of MCI-NP compared with PBS under 808 nm laser irradiation (0.8 W·cm^-2^). **d** Temperature-time profile of MCI-NP under 808 nm laser irradiation at a lower power density (0.33 W·cm^-2^).


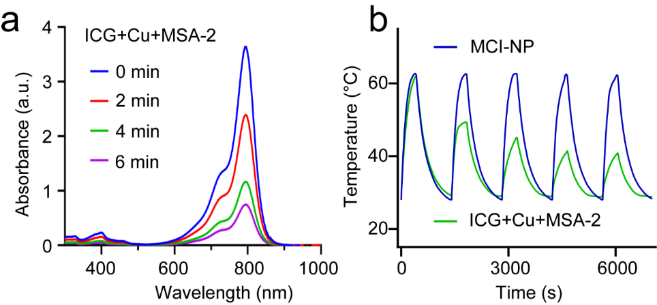


**Fig. S4** Comparison of the photostability and photothermal stability between MCI-NP and a physical mixture of Cu^2+^, MSA-2, and ICG. **a** Changes in UV-vis absorption spectra under continuous 808 nm laser irradiation. **b** Photothermal stability during repeated heating–cooling cycles under 808 nm laser irradiation.


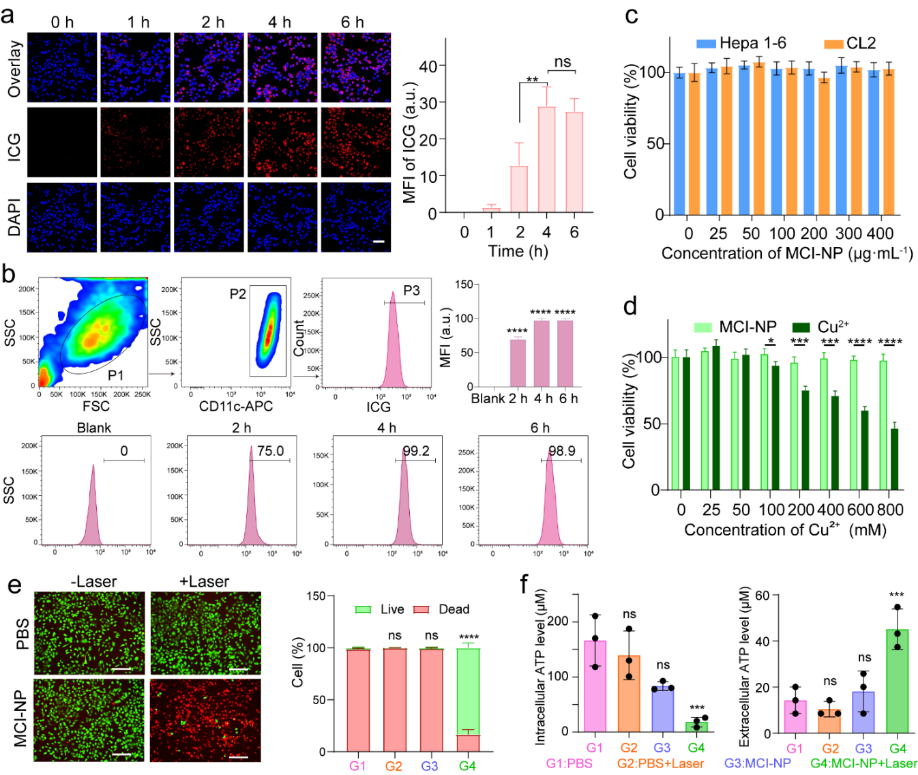


**Fig. S5** Cellular uptake, cytocompatibility, and photothermal effects of MCI-NP in vitro. **a** CLSM images of Hepa1-6 cells incubated with MCI-NP for different durations (0, 1, 2, 4, and 6 h). Intracellular ICG fluorescence intensity increased progressively with incubation time and reached a plateau at 4 h, indicating time-dependent cellular uptake and saturation of MCI-NP internalization. **b** Flow cytometric analysis of MCI-NP uptake by DC2.4 cells. ICG fluorescence intensity was used to quantify nanoparticle internalization, confirming efficient uptake by antigen-presenting cells. **c** Cell viability of Hepa1-6 tumor cells and normal hepatocyte BNL CL.2 cells after incubation with MCI-NP at different concentrations (0-400 μg·mL^-1^), determined by CCK-8 assay. Cell viability remained close to 100% across the tested concentration range, indicating good cytocompatibility of MCI-NP. **d** Cell viability of Hepa1-6 cells after incubation with free Cu^2+^ or MCI-NP at equivalent Cu^2+^ concentrations, determined by CCK-8 assay. MCI-NP exhibited significantly lower cytotoxicity than free Cu^2+^, indicating that nanoparticle assembly mitigates Cu^2+^-associated toxicity. **e** Representative live/dead fluorescence staining images of Hepa1-6 cells after different treatments, including PBS, MCI-NP alone, laser irradiation alone, and MCI-NP combined with laser irradiation (808 nm, 0.8 W·cm^-2^, 10 min). Live cells were stained with calcein-AM (green), and dead cells were stained with propidium iodide (PI, red). Scale bar: 100 μm. **f** Intracellular and extracellular ATP levels in Hepa1-6 cells after different treatments. Intracellular ATP content decreased markedly after MCI-NP-mediated PTT, accompanied by a pronounced increase in extracellular ATP release. Data are presented as mean ± SD (n = 3). Statistical significance was determined by one-way ANOVA (****p* < 0.001).


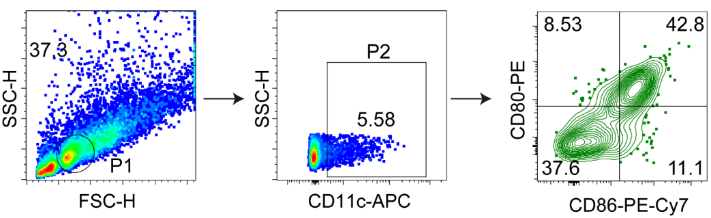


**Fig. S6** Representative gating strategy for flow cytometric analysis of DC maturation (related to Fig. 3e).


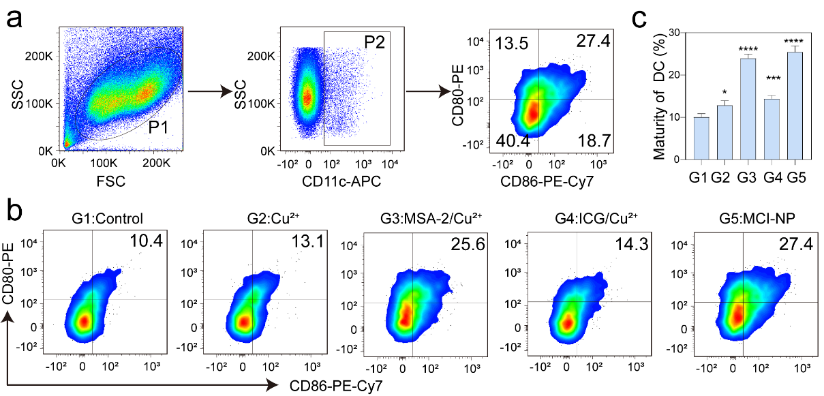


**Fig. S7** Evaluation of the contribution of individual components to DC maturation. **a** Gating strategy for flow cytometric analysis of CD80^+^ CD86^+^ mature BMDCs. **b** Representative flow cytometry plots of DC maturation after treatment with different formulations. **c** Quantification of CD80^+^ CD86^+^ mature DCs (CD11c^+^ gate) in the indicated treatment groups. Data are presented as mean ± SD. Statistics analysis: One-way ANOVA versus PBS control (G1). Significance levels: **p* < 0.05, ****p* < 0.001, *****p* < 0.0001.


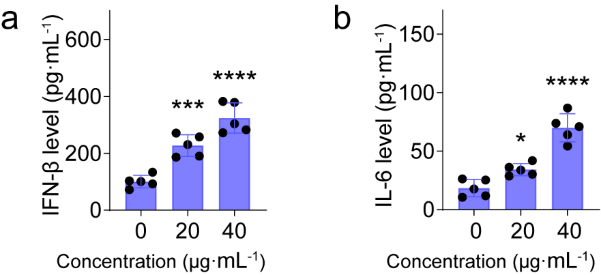


**Fig. S8** ELISA quantification of IFN-β and IL-6 levels in culture supernatants of Hepa1-6 tumor cells following incubation with different concentrations of MCI-NP (n = 5). Statistics: One-way ANOVA, compared with the PBS control group. Data are presented as mean ± SD. Significance levels: **p* < 0.05, ****p* < 0.001, *****p* < 0.0001.


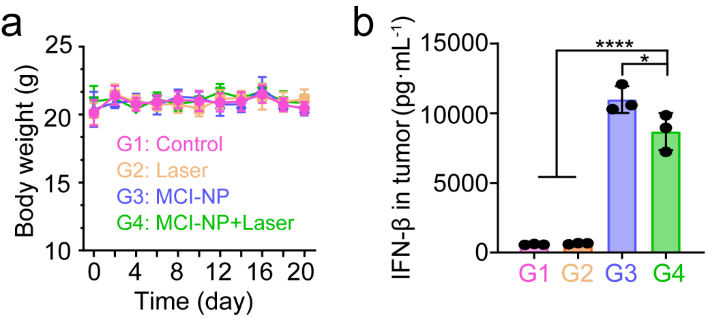


**Fig. S9** **a** Body weight changes of mice in different treatment groups (PBS, Laser, MCI-NP, and MCI-NP+Laser) during the early observation period. No significant body weight loss was observed, indicating acceptable treatment tolerability. **b** Intratumoral IFN-β levels measured by ELISA after different treatments. MCI-NP-based treatments resulted in elevated IFN-β secretion compared with control groups, indicating activation of type I interferon signaling.


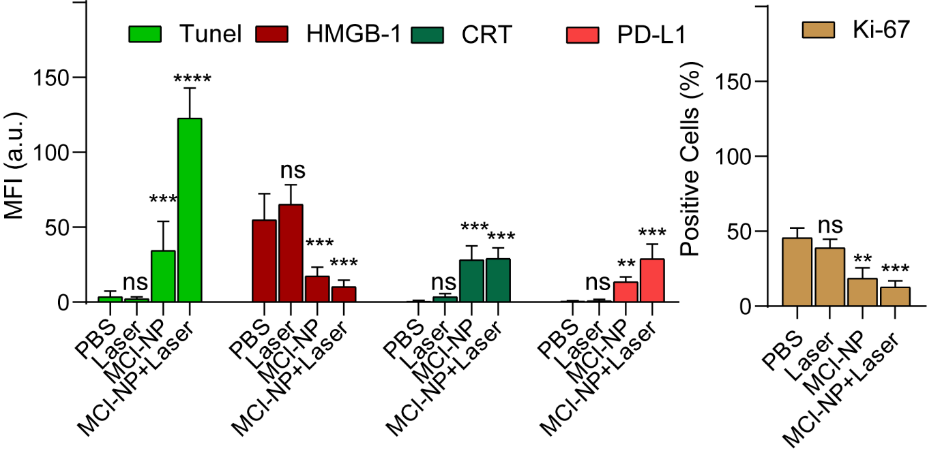


**Fig. S10** Quantitative analysis of tumor cell death and ICD markers following treatment (related to Fig. 4). Fluorescence images of TUNEL, HMGB-1, CRT, and PD-L1 were analyzed using ImageJ software to calculate mean fluorescence intensity (MFI). Immunohistochemistry (IHC) images of Ki67 were quantified to determine the percentage of Ki-67-positive cells. Quantification was performed on three representative fields per sample (n = 3). Data are presented as mean ± SD. Statistical analysis was performed using one-way ANOVA, comparing each treatment group with the PBS control. Significance levels: ***p < 0.01, ***p < 0.001, ****p < 0.0001.*


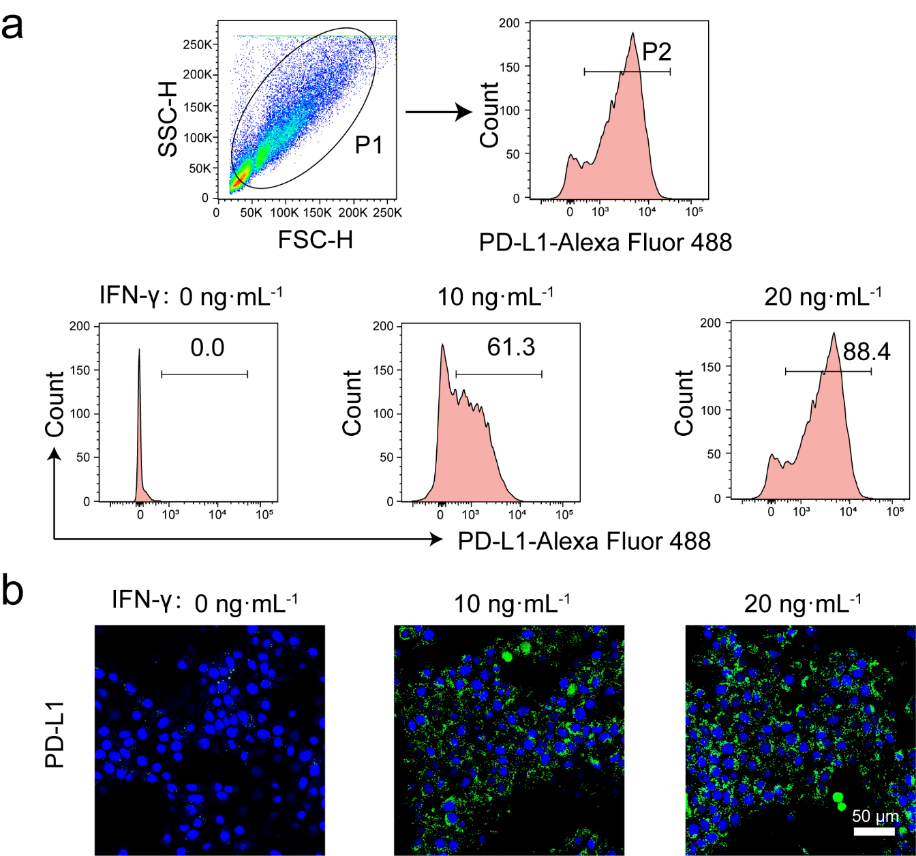


**Fig. S11** Dose-dependent upregulation of PD-L1 in Hepa1-6 cells by IFN-γ. **a** Flow cytometric analysis of PD-L1 expression in Hepa1-6 cells treated with 0, 10, or 20 ng·mL^-1^ IFN-γ. **b** Representative immunofluorescence images showing PD-L1 expression under the same treatment conditions.


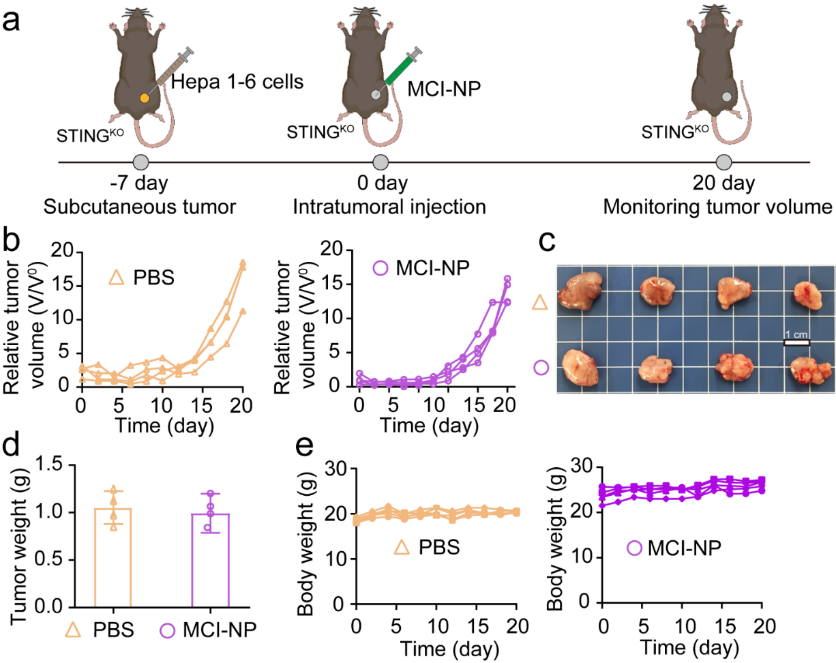


**Fig. S12** Antitumor efficacy of MCI-NP in a STING-knockout (STING-KO) Hepa1-6 subcutaneous tumor model (n = 4). **a** Schematic illustration of the treatment schedule for evaluating MCI-NP in STING-KO mice bearing Hepa1-6 subcutaneous tumors. **b** Relative tumor volume of individual mice following intratumoral administration of PBS or MCI-NP. **c** Representative photographs of excised tumors at the treatment endpoint (day 20). **d** Tumor weights collected at the endpoint (day 20). **e** Body weight changes of individual mice in different treatment groups throughout the observation period. Body weight remained comparable between groups during the study, indicating minimal systemic toxicity associated with MCI-NP treatment.


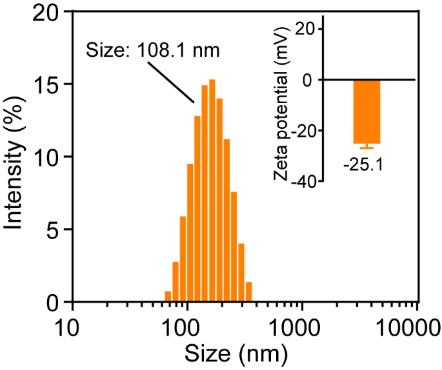


**Fig. S13** DLS analysis of MCI-NP@mPD-1, showing hydrodynamic size distribution and zeta potential.


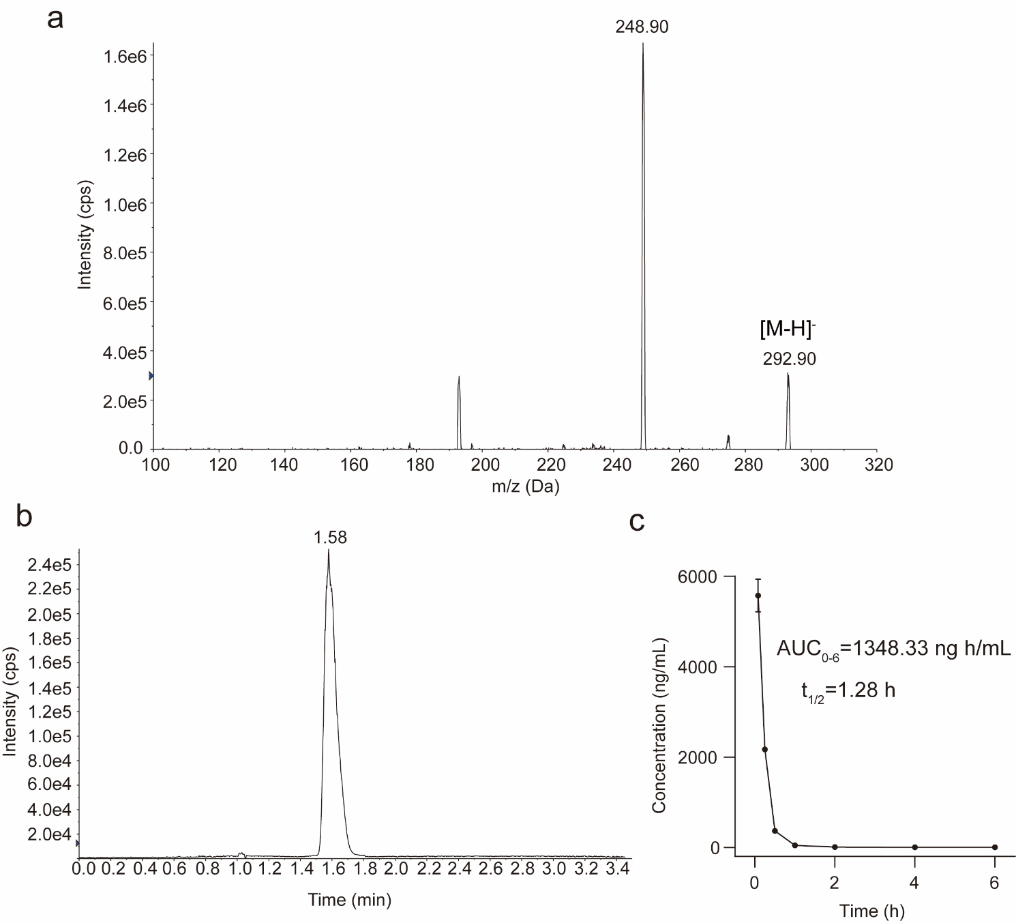


**Fig. S14** LC–MS/MS characterization and plasma pharmacokinetic profile of MSA-2 following intravenous administration of MCI-NP@mPD-1. **a** MS/MS product ion spectrum of MSA-2 detected in negative electrospray ionization mode. The precursor ion was observed at m/z 292.9 as [M−H]⁻. **b** Representative LC-MS/MS chromatogram of MSA-2 in mouse plasma, showing a retention time of 1.58 min. **c** Plasma concentration-time profile of MSA-2, used as a representative analyte for pharmacokinetic evaluation of MCI-NP@mPD-1, following intravenous administration at a dose of 5 mg·kg^-1^. Pharmacokinetic parameters were calculated by non-compartmental analysis. MSA-2 was undetectable at 12 and 24 h post-injection, and these data points were excluded from curve fitting.


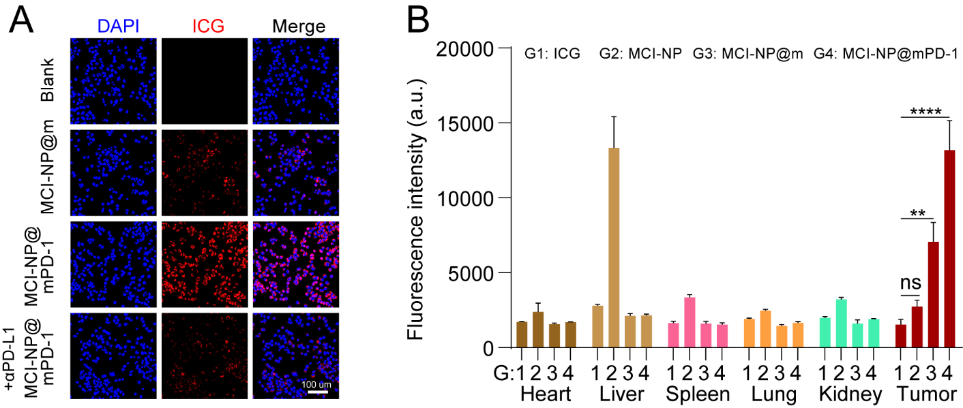


**Fig. S15** **a** CLSM images of Hepa1-6 cells pretreated with IFN-γ to induce PD-L1 expression after incubation with membrane-coated nanoparticles lacking PD-1 (MCI-NP@m) or PD-1 membrane-camouflaged nanoparticles (MCI-NP@mPD-1). Scale bars, 100 μm. **b** Semi-quantitative analysis of *ex vivo* fluorescence intensities in major organs (heart, liver, spleen, lung, and kidney) and tumors harvested at 36 h post-injection following intravenous administration of free ICG, MCI-NP, MCI-NP@m, or MCI-NP@mPD-1 (n = 3). Fluorescence signals were quantified using ImageJ. Statistics: One-way ANOVA for (b). Data are presented as mean ± SD. Significance levels: ***p* < 0.01, *****p* < 0.0001. Related to Fig. 5f.


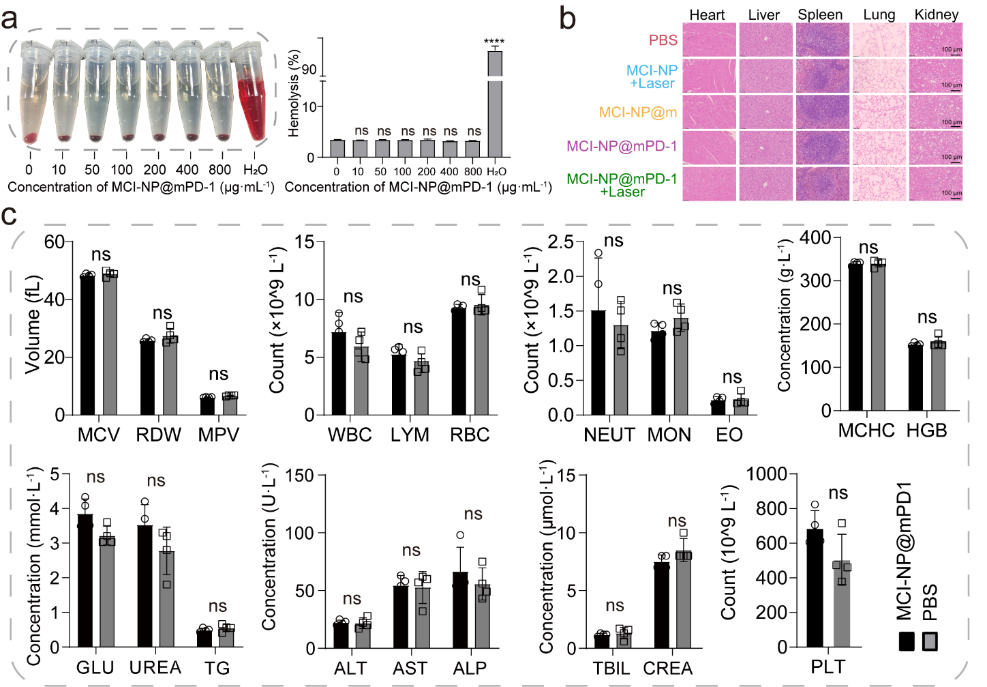


**Fig. S16** Biocompatibility and biosafety evaluation of MCI-NP@mPD-1. **a** Hemolysis assay of MCI-NP@mPD-1 following incubation with murine red blood cells at concentrations ranging from 10 to 800 μg·mL^-1^. No apparent hemolytic activity was observed across the tested concentration range, and quantitative analysis showed that the hemolysis rates of all samples remained below the commonly accepted safety threshold of 5%, indicating good blood compatibility. **b** H&E staining of major organs (heart, liver, spleen, lung, and kidney) collected from treated mice. Representative histological images revealed intact tissue architecture without evident inflammatory infiltration, necrosis, or pathological abnormalities, further confirming the *in vivo* biosafety of MCI-NP@mPD-1. Scale bars, 100 μm. **c** Hematological and serum biochemical analysis of mice after intravenous administration of MCI-NP@mPD-1. Liver and kidney function markers, as well as blood routine parameters, showed no statistically significant differences compared with control group (p > 0.05), suggesting no detectable systemic toxicity or metabolic disturbance.


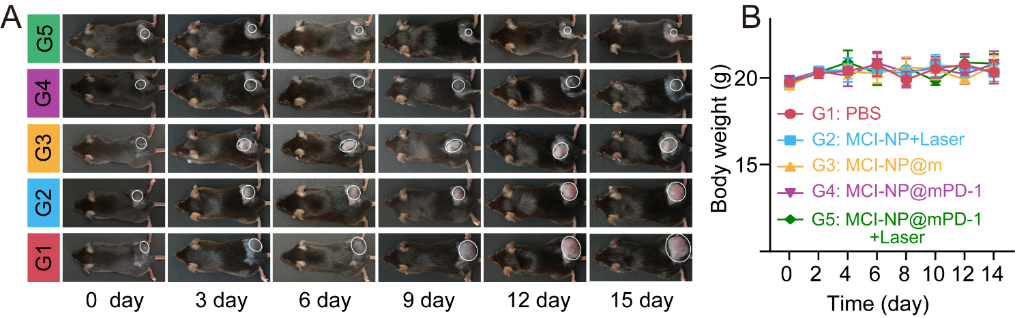


**Fig. S17** Representative tumor images and body weight monitoring during treatment. Related to Fig. 5. **a** Representative photographs of tumor-bearing mice from each treatment group acquired at the indicated time points during the observation period. One mouse per group is shown. **b** Body weight changes of mice in different treatment groups throughout the treatment and observation period, indicating no significant systemic toxicity. Data are presented as mean ± SD.


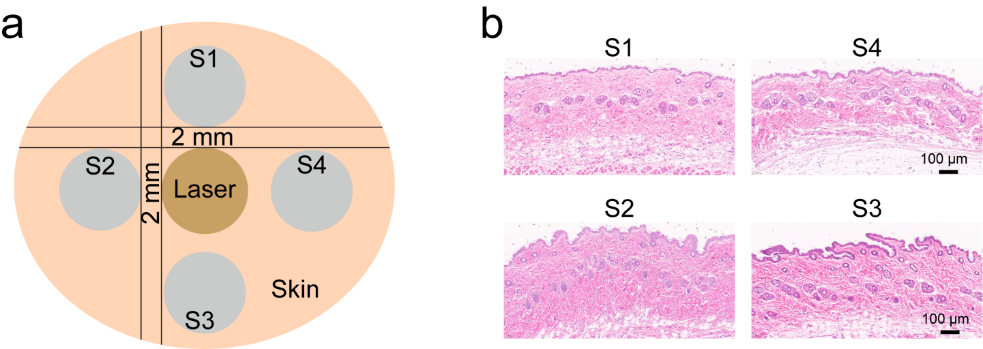


**Fig. S18** H&E staining of normal skin tissues collected approximately 2 mm from the tumor margin at 24 h after photothermal treatment in MCI-NP@mPD-1+Laser-treated mice. Scale bars, 100 μm.


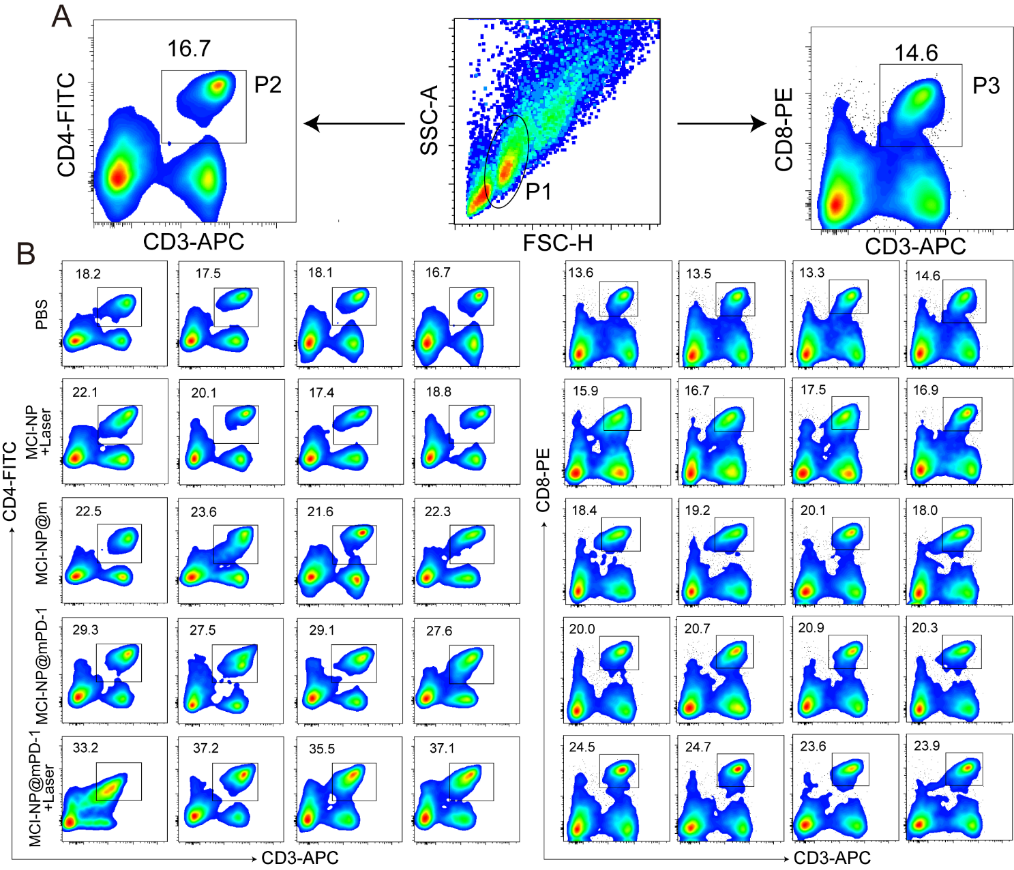


**Fig. S19** Representative gating strategy and individual flow cytometry plots for analysis of intratumoral CD3^+^ CD4^+^ and CD3^+^ CD8^+^ T cells (related to Fig. 6b).


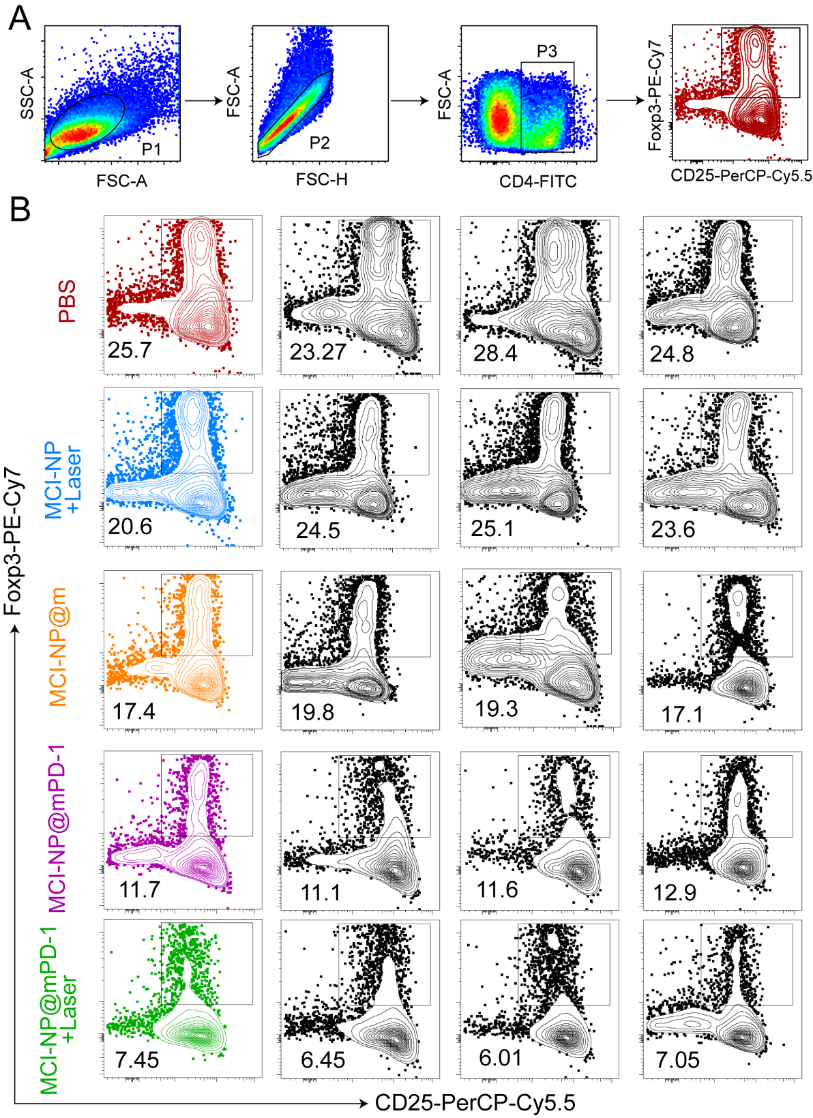


**Fig. S20** Representative gating strategy and individual flow cytometry plots for analysis of intratumoral Tregs (related to Fig. 6b).


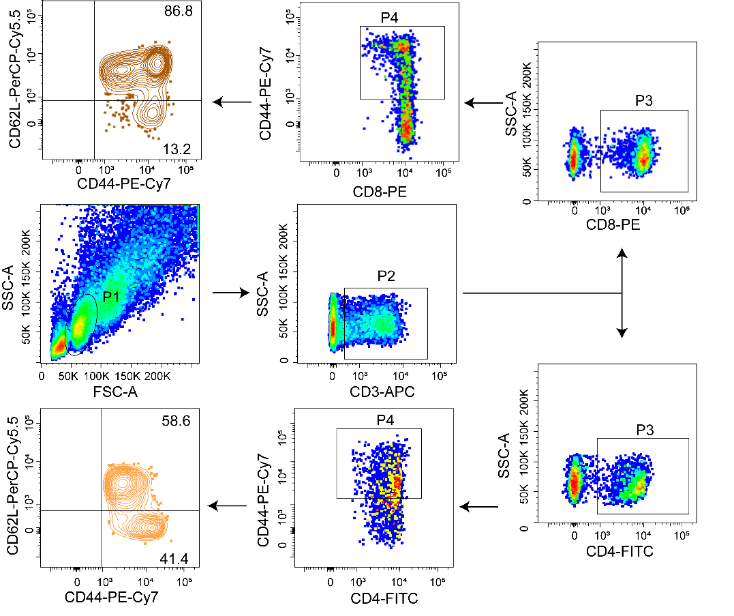


**Fig. S21** Representative gating strategy for flow cytometric analysis of effector memory T cells (T_EM_; CD44^+^ CD62L^−^) in peripheral blood (related to Fig. 7g).


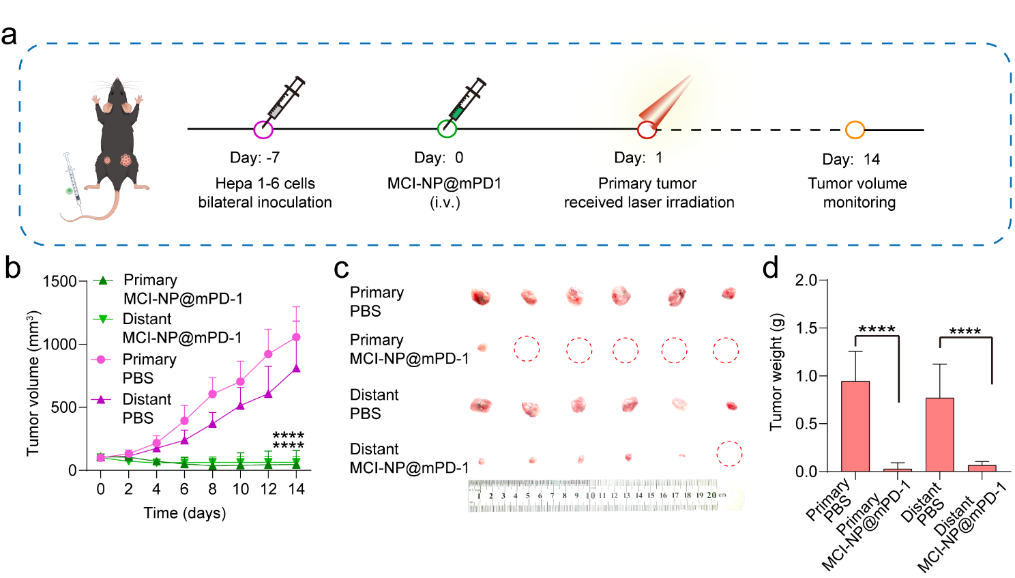


**Fig. S22** Antitumor efficacy of MCI-NP@mPD-1-mediated photothermal-immunotherapy in a bilateral Hepa1-6 tumor model. **a** Schematic illustration of the experimental design. Mice bearing bilateral Hepa1-6 tumors were intravenously administered MCI-NP@mPD-1, followed by laser irradiation applied only to the primary tumor. **b** Growth curves of primary and distant tumors in PBS and MCI-NP@mPD-1+Laser groups. **c** Photographs of excised primary and distant tumors collected at the experimental endpoint. **d** Final weights of primary and distant tumors from each treatment group. Data are presented as mean ± SD (n = 6). Statistical significance was determined by unpaired two-tailed Student’s t-test. *****p* < 0.0001.

**Supplementary References**

1. M. Wu, C. Zhang, A. Zhong, H. Li, Y. Zhou et al., Systemic delivery of stealth adenoviral vector to reinvigorate antitumor immunity by PD-L1 genome editing and TIGIT/CD155 blockade. Fundam. Res. (2025). <https://doi.org/10.1016/j.fmre.2025.04.015>
2. W. Peng, Y. Cao, Y. Zhang, A. Zhong, C. Zhang et al., Optimal Irreversible Electroporation Combined with Nano‐Enabled Immunomodulatory to Boost Systemic Antitumor Immunity. Adv. Healthcare Mater. **13**(7), 2302549 (2024). <https://doi.org/10.1002/adhm.202302549>
3. Y. Zhang, A. Zhong, J. Min, H. Tu, Y. Cao et al., Biomimetic responsive nanoconverters with immune checkpoint blockade plus antiangiogenesis for advanced hepatocellular carcinoma treatment. ACS Appl. Mater. Interfaces **16**(6), 6894-6907 (2024). <https://doi.org/10.1021/acsami.3c18140>
